# Pro-inflammatory and pro-resolving lipid mediators of inflammation in HIV: effect of aspirin intervention

**DOI:** 10.1016/j.ebiom.2023.104468

**Published:** 2023-02-13

**Authors:** Jesmond Dalli, Douglas Kitch, Meagan P. O'Brien, Peter W. Hunt, Nicholas Funderburg, Daniela Moisi, Amita Gupta, Todd T. Brown, Phyllis C. Tien, Judith A. Aberg, Rupak Shivakoti

**Affiliations:** a William Harvey Research Institute, Barts and the London School of Medicine and Dentistry, Queen Mary University of London, London, UK; b Center for Inflammation and Therapeutic Innovation, Queen Mary University of London, London, UK; c Center for Biostatistics in AIDS Research, Harvard T.H. Chan School of Public Health, Boston, USA; d Regeneron Pharmaceuticals Inc., Tarrytown, USA; e Department of Medicine, University of California, San Francisco School of Medicine, USA and Department of Veterans Affairs Medical Center, San Francisco, USA; f Division of Medical Laboratory Science, School of Health and Rehabilitation Sciences, Ohio State University, Columbus, USA; g Department of Medicine, School of Medicine, Case Western Reserve University, Cleveland, USA; h Department of Medicine, Johns Hopkins School of Medicine, Baltimore, USA; i Department of Medicine, Icahn School of Medicine at Mount Sinai, New York, USA; j Department of Epidemiology, Mailman School of Public Health, Columbia University, New York, USA

**Keywords:** HIV, SPMs, Eicosanoids, Aspirin, Inflammation

## Abstract

**Background:**

Persons with HIV (PWH) have an increased risk of cardiovascular disease (CVD) compared to HIV-seronegative individuals (SN). Inflammation contributes to this risk but the role of lipid mediators, with central roles in inflammation, in HIV infection remain to be established; further aspirin reduces CVD risk in the general population through production of some of these anti-inflammatory lipid mediators, but they have not been studied in PWH.

**Methods:**

We evaluated the relationship between plasma lipid mediators (i.e. 50 lipid mediators including classic eicosanoids and specialized pro-resolving mediators (SPMs)) and HIV status; and the impact of aspirin in PWH on regulating these autacoids. Plasma samples were obtained from 110 PWH receiving antiretroviral therapy (ART) from a randomized trial of aspirin (ACTG-A5331) and 107 matched SN samples (MACS-WIHS Combined Cohort).

**Findings:**

PWH had lower levels of arachidonic acid-derived pro-inflammatory prostaglandins (PGs: PGE_2_ and PGD_2_) and thromboxanes (Tx: TxB_2_), and higher levels of select pro-resolving lipid mediators (e.g. RvD4 and MaR2_n−3 DPA_) compared to SN. At the interval tested, aspirin intervention was observed to reduced PGs and Tx, and while we did not observe an increase in aspirin triggered mediators, we observed the upregulation of other SPM in aspirin treated PWH, namely MaR2_n−3 DPA_.

**Interpretation:**

Together these observations demonstrate that plasma lipid mediators profiles, some with links to systemic inflammation and CVD risk, become altered in PWH. Furthermore, aspirin intervention did not increase levels of aspirin-triggered pro-resolving lipid mediators, consistent with other reports of an impaired aspirin response in PWH.

**Funding:**

10.13039/100000002NIH.

**Research in context**

**Evidence before this study**

Aspirin can reduce cardiovascular disease (CVD) risk in the general population, in part through its anti-inflammatory properties. For example, aspirin is known to reduce levels of pro-resolving (i.e. anti-inflammatory) lipid mediators of inflammation. Persons with HIV (PWH) have higher risk of CVD and aspirin administration could potentially reduce CVD risk in this population. We searched PubMed through August 20, 2022 for studies on “aspirin” AND “HIV” AND “lipid mediators of inflammation” (or variations of these terms), but could not find any study that comprehensively assessed pro-inflammatory and pro-resolving lipid mediators of inflammation in PWH before and after aspirin intervention. Further, we only found one small study (n < 20 per group) that assessed lipid mediator of inflammation profile in PWH.

**Added value of this study**

In a randomized trial of aspirin intervention in 110 PWH, we comprehensively assessed the levels of pro-inflammatory and pro-resolving lipid mediators before and after aspirin intervention. Our results show that, in the interval tested, 12 weeks of aspirin administration did not increase levels of pro-resolving lipid mediators of inflammation in PWH. In addition, utilizing a well-characterized and well-matched cohort of HIV-seronegative individuals, our study also is the largest and most comprehensive study to characterize data on alterations of lipid mediators of inflammation by HIV status.

**Implications of all the available evidence**

Our findings suggest that the lipid mediator of inflammation profile, some with important functions in CVD risk, is distinct by HIV status. Importantly, our data also suggest aspirin does not increase the production of aspirin triggered specialized pro-resolving mediators (AT-SPM) in PWH. These results are in line with other findings that inhibition of platelet activation in response to aspirin is also blunted in PWH. Given the potent platelet directed activities of AT-SPM, we believe that our present observations provide insights into mechanism behind the failure of aspirin to regulate platelet responses in these patients. Taken together, these results suggest that aspirin might potentially be less effective in PWH. The clinical implication of these results for CVD prevention in PWH needs to further addressed in future studies.

## Introduction

Persons with HIV (PWH) have an increased risk of cardiovascular disease (CVD) compared to HIV-seronegative individuals (SN).^1^, ^2^, ^3^ Recent studies have implicated increased inflammation in PWH for this higher CVD risk.^1^^,^^2^^,^^4^ The inflammatory profile in PWH is characterized by increased levels of soluble protein mediators, that are thought to be related to gut barrier dysfunction, monocyte activation and systemic inflammation.^5^, ^6^, ^7^, ^8^, ^9^, ^10^ While initiation of antiretroviral therapy (ART) reduces inflammation, levels remain elevated compared to SN.^5^

**Fig. 1:**
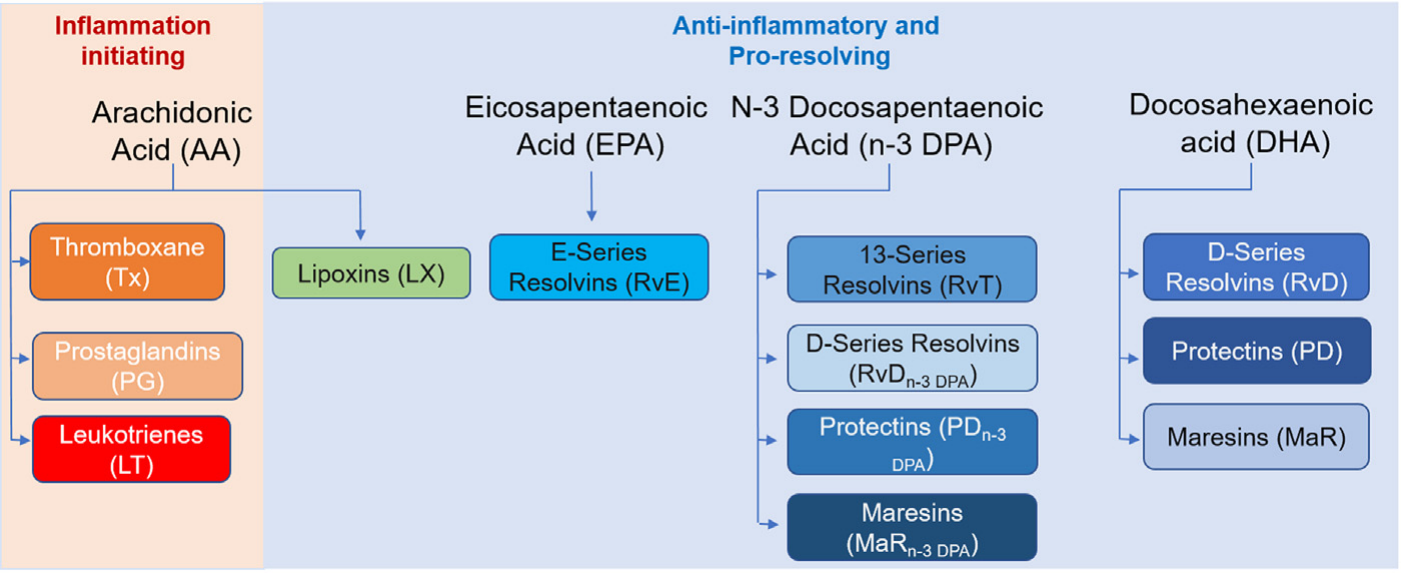
Bioactive lipid families. Illustration summarizing the bioactive mediator families evaluated in the present study together with their parent fatty acid and overall classification of their biological activities.

Despite the critical role that inflammation plays in HIV outcomes such as CVD, the role of lipid mediators of inflammation (Fig. 1 and Supplementary Table S1) in HIV are not well understood. Limited studies have compared levels of the classic eicosanoids derived from arachidonic acid (AA), namely the prostaglandin (PG), leukotriene (LT) and thromboxane (Tx) families,^11^, ^12^, ^13^, ^14^ between PWH and SN individuals. These studies have shown that PWH not receiving ART have higher levels of mediators, including PGE_2_ and TxB_2_, compared to SN.^11^^,^^13^^,^^14^ With ART initiation, levels of these mediators decrease, with some studies showing lower levels even when compared to SN.^12^, ^13^, ^14^

It is now appreciated that lipid mediators mainly from the omega-3 essential fatty acids eicosapentaenoic acid, n−3 docosapentaenoic acid and docosahexaenoic acid and termed as specialized pro-resolving mediators (SPM) are central to the reprogramming of immune responses to limit inflammation.^15^, ^16^, ^17^ These mediators are classified into four main families named resolvins, protectins, maresins and lipoxins (Fig. 1). Recent studies have also implicated SPM in regulating host responses to acute viral infection.^18^, ^19^, ^20^ Data on SPMs are lacking in chronic infections including in PWH. In a small cohort study, we recently found that peripheral blood SPM concentrations are reflective of the immune profile in PWH as well as responsiveness to ART,^21^ but larger studies are needed.

Studies investigating the mechanisms of aspirin in the general population, where it used to reduce CVD risk, demonstrate that, *via* the acetylation of cyclooxygenase (COX)-2, this potent anti-inflammatory drug not only reduces the production of classic eicosanoids, namely PG and Tx, it also upregulates the formation of epimeric forms of several of the SPM (i.e. aspirin-triggered SPM). Aspirin triggered SPM are linked with the regulation of both immune and vascular responses to limit the onset and progression of cardiovascular disease in a range of experimental settings including sickle cell disease, atherosclerosis and neointimal hyperplasia. For example, aspirin triggered LXA_4_ (also referred to as 15R-LXA_4_ and 15-epi-LXA_4_) protects against the development of vascular lesions in murine models of atherosclerosis, decreasing macrophage infiltration into the aorta and downregulating the expression of pro-inflammatory cytokines and chemokines.^22^ Aspirin triggered RvD1 (also referred to as 17R-RvD1) attenuates PDGF-induced vascular smooth muscle cell migration, a mechanism linked with the development of neointimal hyperplasia.^23^ This mediator also protects against inflammatory vasculopathy in experimental sickle cell disease reducing the expression of hemoxiganse-1 in isolated aortas and the expression endothelial activation markers, including of endothelin-1 and vascular cell adhesion molecule 1.^24^ We recently found that this mechanism is linked with the immune-directed activities of aspirin in experimental chronic inflammation.^25^ Although aspirin has been shown to reduce aspects of systemic inflammation and immune activation in healthy volunteers,^26^^,^^27^ aspirin did not reduce biomarkers of HIV-related immune activation in PWH receiving ART in a placebo-controlled trial.^28^ Furthermore, in PWH on ART, the effects of aspirin on the inhibition of platelet activation are impaired.^28^, ^29^, ^30^ Given the attenuated effects of aspirin on platelet activation and immune activation in PWH, we investigated whether the effects of aspirin on SPMs are similarly diminished.

To address some of the gaps in knowledge related to the role of lipid mediators (and their biosynthetic pathways) and in particular SPM in PWH, we compared levels of lipid mediators of inflammation in peripheral blood of PWH on suppressive ART and SN from well-characterized study populations.^28^^,^^31^^,^^32^ We also assessed the relationship of these lipid mediators with protein mediators of inflammation (e.g. monocyte activation marker soluble CD14 that is associated with higher CVD risk) among PWH. Finally, leveraging our randomized controlled trial of aspirin in PWH on suppressive ART,^28^ we also studied the effect of aspirin on these lipid mediators of inflammation.

## Methods

### Study population

For this study, we assessed lipid mediators of inflammation from adult PWH on suppressive ART and from adult SN.

All PWH with available baseline plasma samples (N = 110) from the AIDS Clinical Trials Group (ACTG) A5331 study (NCT02155985) were included in this analysis. The A5331 study, conducted in the United States from August 2014 to March 2015, has been detailed elsewhere.^28^ In brief, this was a three-arm randomized trial of PHW comparing the effects of 12 weeks of daily aspirin at either a 300 mg dose or 100 mg dose to placebo on soluble and cellular markers of inflammation (e.g. monocyte activation). PWH participants were on suppressive ART for at least 48 weeks prior to study initiation. Eligibility criteria is detailed in the Supplement.

Matched adult SN (87 men, 20 women) were selected from a large ongoing cohort study in the United States: The Multicenter AIDS Cohort Study (MACS)^32^—Women's Interagency HIV Study (WIHS)^31^ Combined Cohort Study.^33^ After applying the eligibility criteria of the A5331 study, SN were matched 1:1 to PWH on sex, age, body mass index (BMI), race/ethnicity, smoking status, drinking status and statin use.

### Ethics approval

Study participants provided written informed consent and the study was approved by relevant ethics committees (Supplement).

### Laboratory assessment

#### Targeted lipid mediator profiling

EDTA plasma samples were collected from participants as part of each study (details in Supplement). For lipid mediator profiling, plasma was placed in four volumes of ice-cold methanol and containing deuterium labelled internal standards to facilitate identification and quantification. All samples were profiled using liquid chromatography tandem mass spectrometry (LC-MS/MS) as previously described.^34^

#### Soluble and cellular markers of inflammation

Plasma levels of soluble CD14 (sCD14) was measured at baseline as part of the parent A5331 study using enzyme-linked immunosorbent assays (R&D Systems, Minneapolis, MN).^28^ Measures of other soluble and cellular markers are detailed in the Supplement.

### Statistical analysis

A total of 50 unique lipid mediators were measured in plasma samples, with 42 SPMs and 8 pro-inflammatory lipid mediators (Fig. 1 and Supplementary Table S1). These 50 lipid mediators were also categorized into 12 metabolome families (9 SPMs and 3 pro-inflammatory) (Fig. 1, Supplementary Table S1 and further detailed in the Supplement).

The first objective of this study was to compare plasma levels of i) lipid mediators and ii) metabolomes between PWH (pre-intervention) and SN individuals. To visually examine how mediators/metabolomes and these two populations cluster together, hierarchical clustering heat maps were created for mediators/metabolomes (Supplement). Analysis of covariance (ANCOVA) was used to assess the HIV effect on the 12 metabolomes after adjustment for sex, race/ethnicity, age, BMI, smoking status, drinking status and statin use (Supplement). To control the false discovery rate (FDR) of these 12 analyses, the Benjamini-Hochberg procedure was used and the FDR-adjusted p-values were assessed at the 5% significance level. For the individual mediators, we used supervised partial least squares discriminant analysis (PLS-DA) method, a data reduction method to account for the large numbers of mediators. These identified mediators, those with “above average importance” based on Variable importance for the projection (VIP) values >1, were each used in exploratory ANCOVA models (Supplement).

The second objective of this study was to determine the association of lipid mediators of inflammation with the monocyte activation marker sCD14 among PWH (i.e. A5331 study participants at baseline). Logistic regression was used to determine the association of high inflammation (outcome variable of “high” sCD14 defined as the highest quartile (Q4) compared to Q1–Q3) with i) individual metabolome, ii) individual principal components (derived from unsupervised principal components analysis) of the metabolome, and iii) individual principal components of the mediators (further details in Supplement). The association of i) individual metabolomes with “high” sCD14 used FDR-adjusted p-values assessed at the 5% significance level.

The final objective of this study was to study whether and how the administration of aspirin changed levels of lipid mediators among PWH (i.e. among A5331 participants pre- and post-intervention). We conducted analysis of variance (ANOVA) to assess the effect of each aspirin arm in A5331 (100 mg and 300 mg arms) relative to placebo on 12-week change (i.e. change in lipid mediators/metabolomes from baseline to 12 weeks) in mediators and metabolomes (further details in Supplement).

With the exception of analyses utilizing FDR-adjusted p-values, all other exploratory analyses used unadjusted p-values conservatively assessed at the 1% significance level to limit the number of false positive findings.

### Role of the funding source

The study sponsors did have a role in the study design; in the collection, analysis, and interpretation of data; in the writing of the report; and in the decision to submit the paper for publication.

## Results

### Study population characteristics

Our study population (N = 217) included 110 PWH on suppressive ART and 107 SN. As expected with the matching of PWH and SN cohorts, study population characteristics including age, sex, race/ethnicity and body max index were similar by HIV status (Table 1). Among PWH, the median (interquartile range) CD4+ T cell count was 608 (465, 776) cells/mm^3^ and 97% had undetectable viral loads. 28% of the PWH were on integrase-inhibitor based regimens, 40% on NNRTI-based and 33% on PI-based regimen.^28^

**Table 1:** Study population baseline characteristics by cohort.

Characteristic	Cohort			
	PWH (N = 110)	SN (N = 107)	Total (N = 217)	p-value
Sex				
Male	88 (80%)	87 (81%)	175 (81%)	0.86^a^
Female	22 (20%)	20 (19%)	42 (19%)	
Age (years)				
Mean (s.d.)	48.0 (10.7)	47.9 (11.0)	47.9 (10.8)	0.92^b^
Median (Q1, Q3)	49.5 (42.0, 55.0)	49 (40, 56)	49 (41, 56)	
Min, Max	21, 72	22, 70	21, 72	
Race/ethnicity				
White Non-Hispanic	57 (52%)	55 (51%)	112 (52%)	0.98^a^
Black Non-Hispanic	35 (32%)	33 (31%)	68 (31%)	
Hispanic (regardless of race)	17 (15%)	18 (17%)	35 (16%)	
Asian, Pacific Islander	1 (1%)	1 (1%)	2 (1%)	
BMI (kg/m^2^)				
Mean (s.d.)	27.1 (5.5)	26.3 (4.4)	26.7 (5.0)	0.53^b^
Median (Q1, Q3)	26.0 (23.3, 29.7)	25.4 (23.3, 28.5)	25.8 (23.3, 29.2)	
Min, Max	16.7, 43.5	18.5, 39.8	16.7, 43.5	
# Missing	2	21	23	
Underweight (<18.5)	3 (3%)	0 (0%)	3 (1%)	0.33^a^
Normal weight (18.5–24.9)	38 (35%)	40 (38%)	78 (36%)	
Overweight/obese ( ≥ 25.0)	67 (62%)	66 (62%)	133 (62%)	
# Missing	2	1	3	
Smoking status				
Never or previously	86 (78%)	83 (78%)	169 (78%)	1.00^a^
Currently	24 (22%)	24 (22%)	48 (22%)	
Alcohol use				
0 or <3 drinks/day	103 (95%)	103 (96%)	206 (96%)	1.00^a^
3+ drinks/day	5 (5%)	4 (4%)	9 (4%)	
Unknown	2	0	2	
Statin use				
No	92 (84%)	91 (85%)	183 (84%)	0.85^a^
Yes	18 (16%)	16 (15%)	34 (16%)	
Fish oil supplement				
No	98 (89%)	87 (100%)	185 (94%)	0.001^a^
Yes	12 (11%)	0 (0%)	12 (6%)	
# Missing	0	20	20	

BMI: body mass index.

a Fisher's Exact Test.

b Wilcoxon Test.

### Lipid mediator profile in PWH and SN individuals

To understand the profile of pro-inflammatory and pro-resolving lipid mediators in PWH (i.e. baseline: pre-aspirin intervention) and SN individuals, we measured plasma levels of 50 lipid mediators belonging to 12 lipid mediator families derived from four major fatty acids namely docosahexaenoic acid (DHA), n−3 docosapentaenoic acid (n−3 DPA), eicosapentanoic acid (EPA) and arachidonic acid (AA) (Fig. 1; median values in Supplementary Tables S2 and S3). In these peripheral blood samples, we identified mediators from each of these families including the DHA and n−3 DPA derived resolvins, protectins and maresins as well as the AA-derived Tx, PG and LT.

**Fig. 2:**
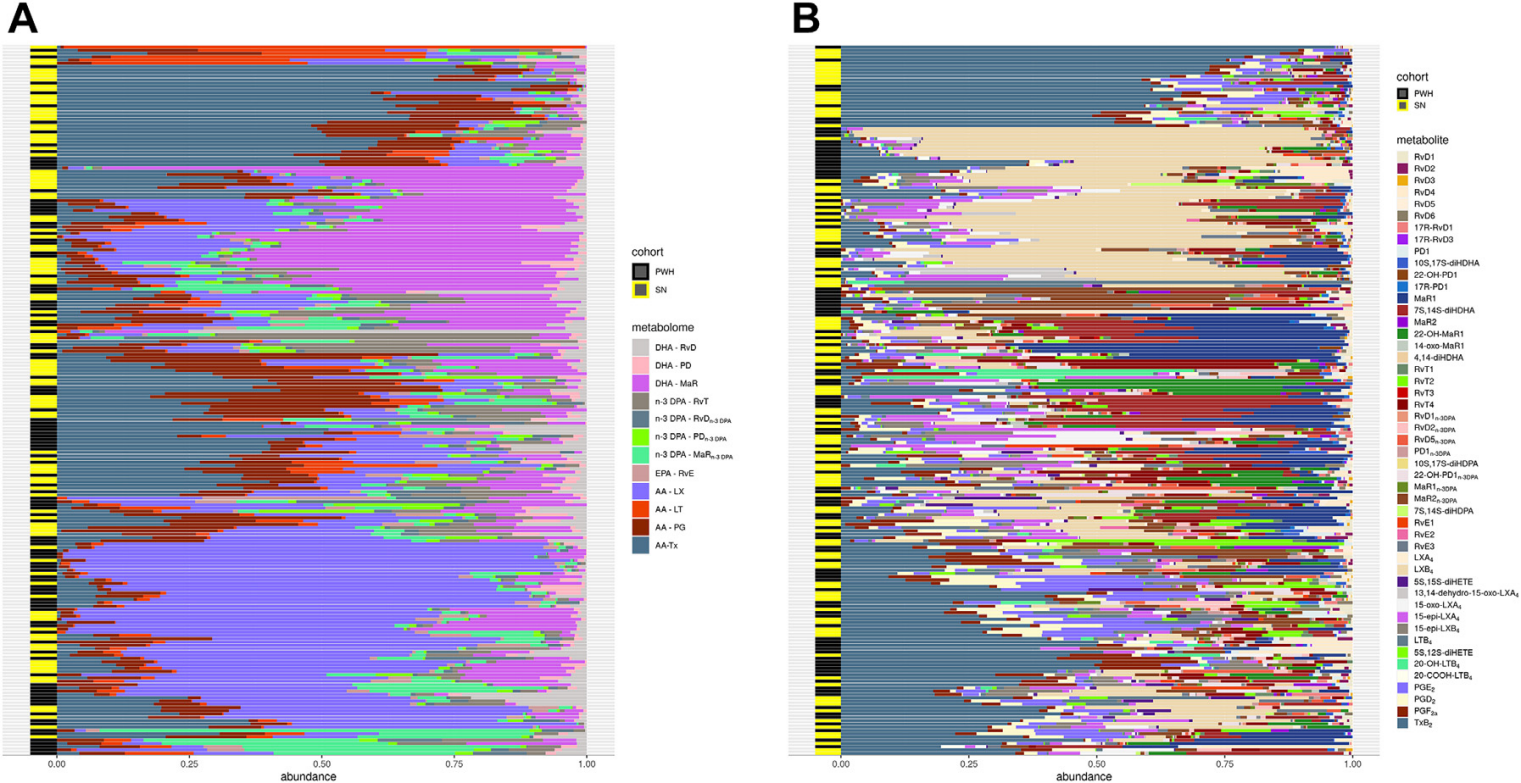
Lipid metabolome and mediator abundance bar plot. A: Metabolome Abundance bar plot. B: Lipid mediator abundance bar plot. Abundance heat map for metabolome (A, left panel) and lipid mediator (B, right panel) for all individuals (N = 217) are shown as a stacked bar graph. Metabolome and individual mediators were log_10_ transformed and standardized. Color coding shows HIV status as well as the metabolome/mediator group.

**Supplementary Fig S1:**
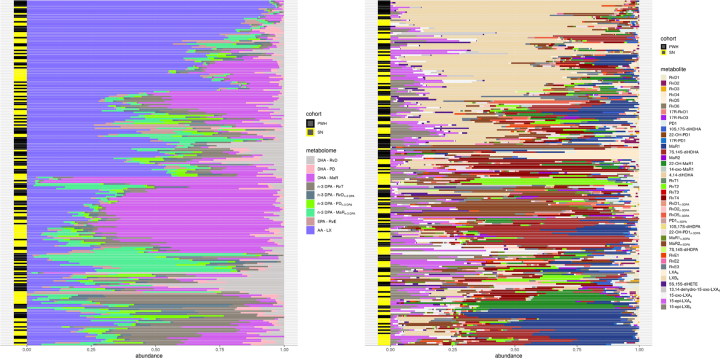


**Supplementary Fig S2:**
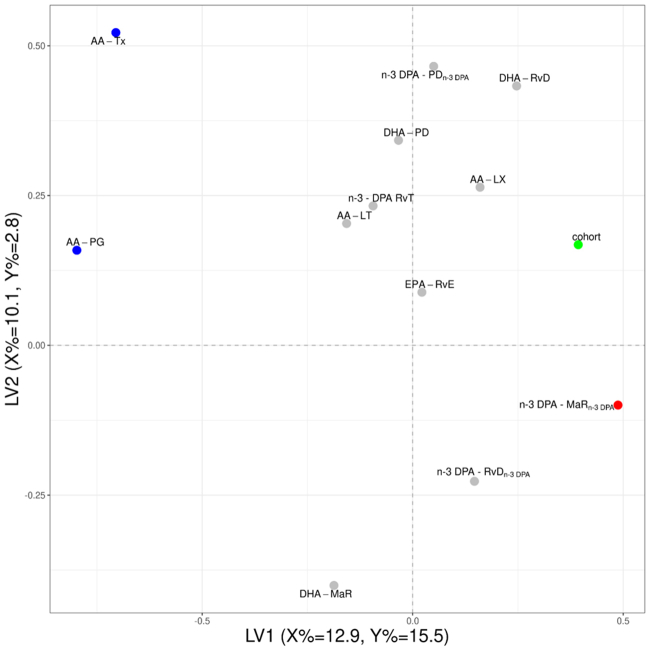


**Supplementary Fig S3:**
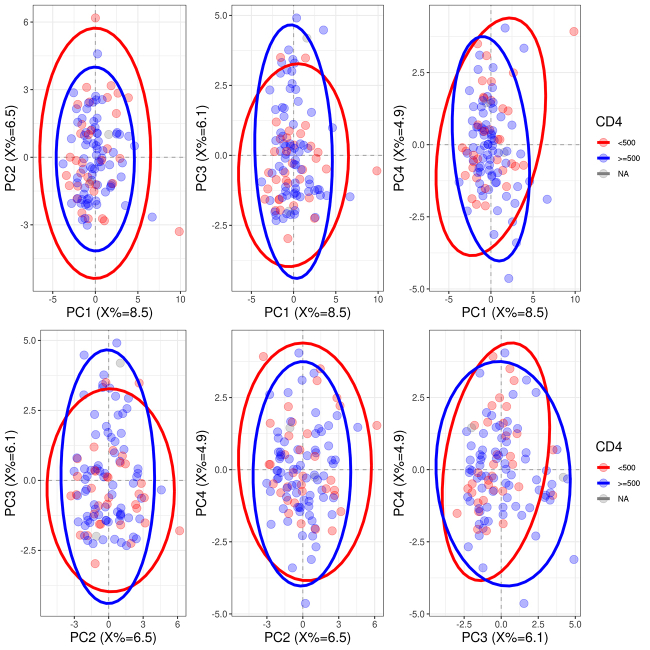


**Supplementary Fig S4:**
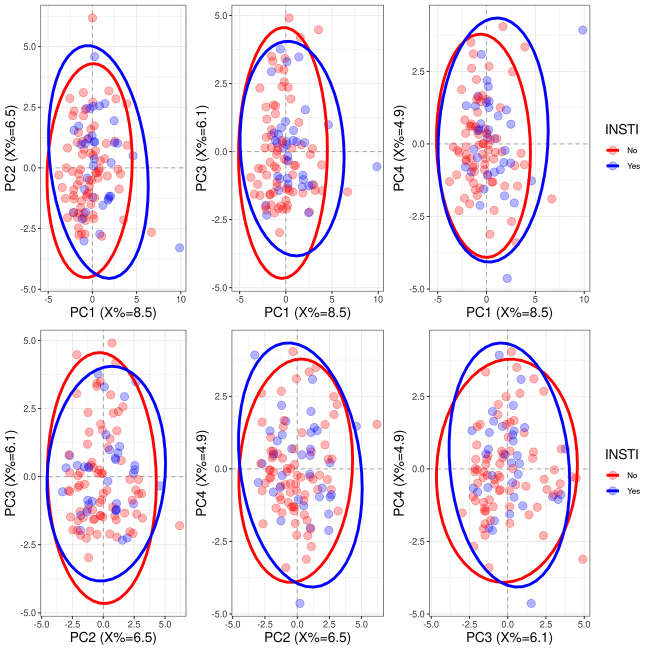


**Supplementary Fig S5:**
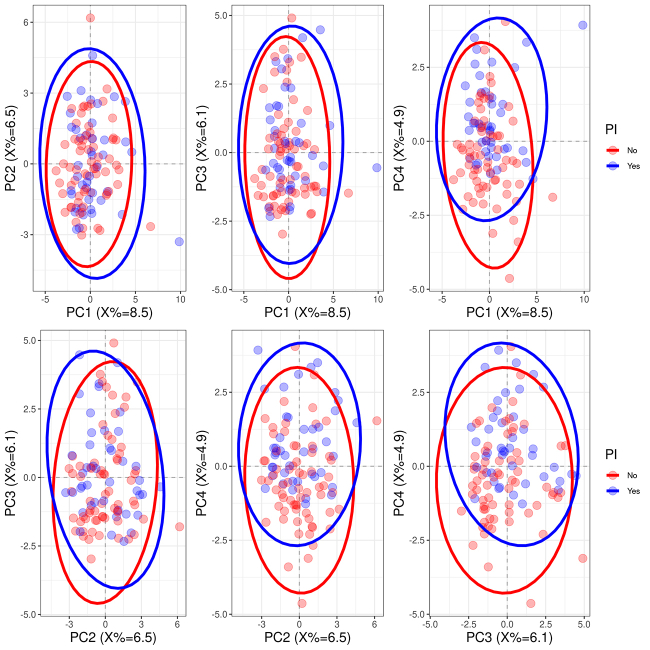


Abundance heatmaps of the overall population (PWH and SN combined) at the lipid mediator family level, showed that while there were differences among individuals, there was no clear clustering by HIV status at either the metabolome or individual mediator level (Fig. 2), with similar observations when we focused on SPM families and mediators (Supplementary Fig. S1). We next conducted ANCOVA analyses to determine the association of HIV status with each of the 12 metabolomes. After adjusting for sex, race/ethnicity, age, body mass index, smoking, heavy drinking and statin use, PWH had lower levels of AA-derived PG (fold difference (Δ) (95% confidence intervals (CI)) = 0.43 (0.31, 0.59); FDR-adjusted p_FDR_<0.001) and Tx (Δ = 0.43 (0.24, 0.78); p_FDR_ = 0.032), and higher levels of MaRn-3 DPA (Δ = 1.48 (1.11, 1.98); p_FDR_ = 0.032) compared to SN (Table 2). Results from PLS-DA also confirmed that AA-derived PG and Tx, and MaR_n−3 DPA_ were the metabolomes most strongly associated with HIV status (Supplementary Fig. S2). Of note, removing 12 individuals on fish oil from the analysis did not change the overall results. Among PWH, there were also no overall differences by ART status or CD4 count (Supplementary Figs. S3–S5).

**Table 2:** Differences in metabolomes by HIV status.

Metabolome outcome	Observations used	PWH geometric LS mean (95% CI)	SN geometric LS mean (95% CI)	Fold difference^a^ (95% CI)	Raw p-value	FDR-adjusted p-value
DHA–RvD (pg/mL)	212	1.43 (1.09, 1.88)	0.97 (0.74, 1.27)	1.49 (1.01, 2.18)	0.044	0.13
DHA–PD (pg/mL)	212	0.55 (0.42, 0.72)	0.55 (0.42, 0.72)	0.99 (0.68, 1.46)	0.98	0.98
DHA–MaR (pg/mL)	212	3.27 (2.26, 4.72)	5.22 (3.61, 7.54)	0.63 (0.37, 1.06)	0.079	0.16
n−3 DPA–RvT (pg/mL)	212	1.52 (1.14, 2.02)	1.63 (1.22, 2.16)	0.93 (0.62, 1.40)	0.74	0.92
n−3 DPA–RvD_n−3 DPA_ (pg/mL)	212	0.56 (0.40, 0.77)	0.52 (0.37, 0.72)	1.07 (0.68, 1.70)	0.76	0.92
n−3 DPA–PD_n−3 DPA_ (pg/mL)	212	0.49 (0.37, 0.63)	0.40 (0.31, 0.52)	1.22 (0.84, 1.76)	0.30	0.50
n−3 DPA–MaR_n−3 DPA_ (pg/mL)	212	2.47 (2.02, 3.03)	1.67 (1.36, 2.05)	1.48 (1.11, 1.98)	0.008	**0.032**
EPA–RvE (pg/mL)	212	0.27 (0.18, 0.39)	0.27 (0.18, 0.40)	0.99 (0.57, 1.73)	0.97	0.98
AA–LX (pg/mL)	212	15.83 (11.65, 21.52)	10.71 (7.88, 14.56)	1.48 (0.96, 2.28)	0.079	0.16
AA–LT (pg/mL)	212	0.66 (0.46, 0.96)	0.86 (0.59, 1.24)	0.77 (0.46, 1.31)	0.34	0.50
AA–PG (pg/mL)	212	4.28 (3.42, 5.36)	10.02 (8.00, 12.55)	0.43 (0.31, 0.59)	<0.001	**<0.001**
AA–Tx (pg/mL)	212	4.18 (2.75, 6.34)	9.67 (6.37, 14.67)	0.43 (0.24, 0.78)	0.006	**0.032**

Analysis of covariance was conducted to assess the differences in metabolomes by HIV status. All models are adjusted for sex, race/ethnicity, age, body mass index, smoking, drinking and statin use. FDR: false discovery rate.

a PWH Geometric LS Mean/SN Geometric LS Mean.

Based on ANCOVA analysis, adjusted for covariates, of 16 metabolites with VIP > 1, PWH had lower levels of 4S,14S-diHDHA (Δ = 0.77 (0.64, 0.93); unadjusted p_unadj_ = 0.006), and various prostanoids including PGE_2_ (Δ = 0.45 (0.30, 0.69); p_unadj_ < 0.001), PGD_2_ (Δ = 0.43 (0.30, 0.61); p_unadj_ < 0.001), PGF_2a_ (Δ = 0.48 (0.33, 0.69); p_unadj_ < 0.001) and TxB_2_ (Δ = 0.43 (0.24, 0.78); p_unadj_ = 0.006), and higher levels of various SPMs including RvD4 (Δ = 2.04 (1.47, 2.82); p_unadj_ < 0.001), MaR2_n-3 DPA_ (Δ = 1.76 (1.25, 2.49); p_unadj_ = 0.001) and RvE2 (Δ = 1.14 (1.03, 1.25); p_unadj_ = 0.008) (Supplementary Table S4).

### Relationship of lipid mediators with protein mediators among PWH

As PWH with higher levels of the monocyte activation marker soluble CD14 (sCD14) have an increased risk of morbidity and mortality, another objective of this study was to determine the relationship of lipid mediators and sCD14 among PWH.

At the family level, no statistically significant associations with ‘high sCD14’ (defined as those in the highest quartile) were observed after FDR adjustment (Supplementary Table S5). For the analysis of individual mediators, the 50 mediators were further reduced to uncorrelated principal components (PCs) using principal components analysis. Among the four PCs with eigenvalues >1, only the first PC was strongly associated with sCD14 category, with a 1 unit higher PC1 score being associated with 42% greater odds of high sCD14 (OR = 1.42 (1.12, 1.79)). PC1 had high positive loadings of PGs (PGF_2a_, PGD_2_, PGE_2_), TxB_2_, and RvD, and had high negative loadings of MaR2_n−3 DPA_ and RvD5 _n−3 DPA_. Interestingly, RvD5_n−3 DPA_ is associated with improved intestinal integrity and reduced microbial translocation.^35^^,^^36^

While our primary focus was on sCD14, we also explored the relationship of the lipid mediators of inflammation with additional markers including sCD163, IL-6 and CD4 T-cell activation (CD38+HLA-DR+). At the metabolome level, only PG had a strong inverse association with CD4 activation (OR: 0.29 (0.12, 0.71)). We did not observe statistically significant associations of other metabolomes or mediators, and their PCs with these markers (**data not shown**).

### Effect of aspirin treatment on lipid mediators among PWH

PWH were randomized to 12 weeks of either daily aspirin 300 mg, aspirin 100 mg or placebo and self-reported adherence to the intervention was high.^28^ Study population characteristics at baseline were similar between the study arms.^28^ Acetylation of COX-2 by aspirin, in addition to blocking PG and TX formation, triggers the formation of epimeric forms of the RvD, PD and LX.^37^^,^^38^ Thus, we evaluated the impact of the intervention on changes to the levels of metabolomes and individual lipid mediators. ANOVA analyses at the lipid mediator family level showed large differences between the intervention arms and placebo for AA-derived PG and Tx metabolomes. For PG, the mean fold change % difference compared to placebo was −64.7 (−80.6, −35.5) for the 300 mg arm and −69.8 (−83.5, −44.9) in the 100 mg arm (Table 3). As an expected function of aspirin on Tx, the mean fold change % difference of Tx compared to placebo was −94.6 (−98.3, −82.9) for the 300 mg arm and −97.8 (−99.3, −93.0) in the 100 mg arm (Table 3).

**Table 3:** Effect of aspirin on lipid mediator metabolomes among PWH.

Metabolome	Treatment arm	Mean fold Δ (95% CI)	Mean fold Δ % difference (95% CI) vs. placebo	Raw p-value vs. placebo	FDR-adjusted p-value vs. placebo	Mean fold Δ % difference (95% CI) vs. 100 mg	Raw p-value vs. 100 mg
DHA–RvD	300 mg	0.91 (0.49, 1.69)	4.3 (−56.5, 150.1)	0.92	0.99	–	–
	100 mg	0.31 (0.17, 0.57)	−64.8 (−85.3, −15.7)	0.020	0.094		
	Placebo	0.88 (0.47, 1.63)					
DHA–PD	300 mg	1.18 (0.64, 2.17)	105.0 (−14.3, 390.8)	0.11	0.28	–	–
	100 mg	0.58 (0.31, 1.06)	0.5 (−58.0, 140.6)	0.99	0.99		
	Placebo	0.57 (0.31, 1.07)					
DHA–MaR	300 mg	0.96 (0.42, 2.20)	43.1 (−55.9, 364.6)	0.55	0.78	–	–
	100 mg	1.79 (0.78, 4.09)	166.4 (−17.9, 764.9)	0.10	0.28		
	Placebo	0.67 (0.29, 1.55)					
n−3 DPA–RvT	300 mg	0.87 (0.47, 1.64)	10.5 (−54.9, 170.5)	0.83	0.99	–	–
	100 mg	1.03 (0.55, 1.94)	30.9 (−46.5, 220.4)	0.55	0.78		
	Placebo	0.79 (0.42, 1.49)					
n−3 DPA–RvD_n−3 DPA_	300 mg	1.70 (0.87, 3.33)	72.4 (−33.8, 349.2)	0.26	0.48	–	–
	100 mg	1.84 (0.94, 3.60)	86.0 (−28.6, 384.7)	0.20	0.40		
	Placebo	0.99 (0.50, 1.95)					
n−3 DPA–PD_n−3 DPA_	300 mg	0.87 (0.52, 1.45)	−38.6 (−70.2, 26.5)	0.18	0.40	–	–
	100 mg	0.99 (0.60, 1.65)	−29.9 (−65.9, 44.5)	0.33	0.53		
	Placebo	1.42 (0.85, 2.37)					
n−3 DPA–MaR_n−3 DPA_	300 mg	1.17 (0.79, 1.73)	−1.7 (−43.5, 71.2)	0.95	0.99	–	–
	100 mg	0.65 (0.44, 0.96)	−45.5 (−68.7, −5.2)	0.032	0.13		
	Placebo	1.19 (0.80, 1.77)					
EPA–RvE	300 mg	0.98 (0.37, 2.58)	−8.4 (−76.9, 263.4)	0.90	0.99	–	–
	100 mg	1.32 (0.50, 3.48)	23.8 (−68.8, 390.9)	0.76	0.99		
	Placebo	1.07 (0.40, 2.85)					
AA–LX	300 mg	0.43 (0.24, 0.77)	−46.6 (−76.5, 21.8)	0.13	0.32	–	–
	100 mg	0.76 (0.43, 1.36)	−5.8 (−58.6, 114.6)	0.89	0.99		
	Placebo	0.81 (0.45, 1.45)					
AA–LT	300 mg	0.50 (0.25, 1.00)	−59.6 (−85.0, 8.8)	0.073	0.25	–	–
	100 mg	0.74 (0.37, 1.48)	−40.1 (−77.8, 61.6)	0.31	0.53		
	Placebo	1.23 (0.61, 2.50)					
AA–PG	300 mg	0.62 (0.41, 0.95)	−64.7 (−80.6, −35.5)	0.001	**0.005**	17.2 (−35.6, 113.0)	0.60
	100 mg	0.53 (0.35, 0.81)	−69.8 (−83.5, −44.9)	<0.001	**0.001**		
	Placebo	1.77 (1.15, 2.71)					
AA–Tx	300 mg	0.10 (0.04, 0.23)	−94.6 (−98.3, −82.9)	<0.001	**<0.001**	145.6 (−22.4, 677.3)	0.13
	100 mg	0.04 (0.02, 0.09)	−97.8 (−99.3, −93.0)	<0.001	**<0.001**		
	Placebo	1.87 (0.82, 4.28)					

The mean fold-change and mean fold-change percent from baseline is shown for the three study arms for each lipid mediator metabolome. Analysis of variance were conducted to test the effect of the 100 mg and 300 mg dose of aspirin as compared to the placebo.

We then repeated the above analyses for the individual lipid mediators. The difference noted above at the metabolome level for PG was mostly driven by PGE_2_ with a mean fold change % difference of −85.5 (−92.6, −71.5) for the 300 mg arm and −83.1 (−91.4, −66.8) for the 100 mg arm (Supplementary Table S6). Differences were also noted for PGD_2_, LTB_4_ and MaR_2_ as noted in Supplementary Table S6. While the TxB_2_ levels were reduced with aspirin as expected, interestingly there were no significant increases in any of the aspirin-triggered SPM mediators: 17R-RvD1, 17R-RvD3, 17R-PD1, 15-epi-LXA_4_ and 15-epi-LXB_4_ (Supplementary Table S6); there were also no increases of these SPMs in the aspirin arms compared to their own baseline.

## Discussion

In this study, we assessed the relationship between circulating lipid mediators of inflammation and resolution with HIV status, monocyte activation and aspirin treatment. When compared with SN, PWH on suppressive ART had lower levels of Tx and various PGs, and higher MaR_n−3 DPA_ and RvD. Interestingly, PWH with higher positive loadings of Tx and PGs and negative loadings of MaR2_n−3 DPA_ and RvD5_n−3 DPA_ (associated with reduced intestinal permeability) had greater odds of having higher sCD14, a monocyte activation marker associated with CVD. After randomization, while aspirin treatment for 12 weeks in PWH was associated with expected decreases in Tx and PG, we did not observe increases in any of the aspirin-triggered SPMs compared to PWH not taking aspirin. Overall, our results show a distinct profile of lipid mediators in PWH compared to SN; and show that aspirin treatment did not increase plasma aspirin-triggered SPMs, consistent with our findings from other studies showing attenuated effects of aspirin on platelets and immune activation in PWH.

In our comparison between PWH on ART and SN, we observed lower levels of AA-derived PG and Tx in PWH, with TxB_2,_ PGE_2_, PGD_2_ and PGF_2a_ being the individual mediators that were significantly lower. Our findings are consistent with most,^12^^,^^13^ but not all published reports.^14^ Most have shown lower levels in circulation of various prostanoids including TxB_2_ and PGE_2_ in PWH on ART compared to uninfected controls. Decreases in overall levels of their precursor polyunsaturated fatty acid (PUFA) in PWH compared to SN observed in other studies^39^^,^^40^ only partly explain these results, as our data shows an increase in the SPMs (e.g. MaR2_n−3 DPA_, RvD4 and RvE4) derived from the SPM precursors (EPA, DHA and n−3 DPA). These SPMs have anti-inflammatory properties; for example, MaR2_n−3 DPA_ reduces neutrophil recruitment and improves macrophage phagocytosis, ultimately protecting against inflammation-mediated organ injury.^41^ These results of lipid mediators are a contrast to the protein soluble pro-inflammatory markers, which are increased in PWH. We hypothesize that the increased levels of these SPMs might partly be a negative feedback to the increased protein and cellular pro-inflammatory profile observed in HIV.^5^

Our findings related to sCD14 and lipid mediators showed relationships among PWH on suppressive ART. sCD14 had positive associations with various PGs and TxB_2_. These results suggest that among PWH on suppressive ART, the pro-inflammatory protein (i.e. sCD14) and lipid mediators are correlated. The results related to SPMs were more complex. Certain SPMs, especially MaR2_n−3 DPA_ and RvD5_n−3 DPA_ gave negative correlations, while other SPMs such as RvD gave positive correlations with sCD14. The observation that RvD5_n−3 DPA_ was negatively correlated with sCD14 may reflect that observed increased microbial translocation in HIV^42^ given that mediator RvD5_n−3 DPA_ was recently linked with, including causally in animal models, the maintenance of intestinal barrier integrity.^35^^,^^36^ Whether increasing levels of RvD5_n−3 DPA_ in PWH individuals can improve intestinal integrity, reduce monocyte activation and inflammation should be tested in future studies.

After aspirin intervention, we observed expected large reductions in levels of PGs and Tx with both doses of aspirin. Aspirin binds and acetylates the cyclooxygenase (COX)-2 enzyme active site, leading to blocking of PG and Tx production.^15^ However, the acetylated COX-2 enzyme and other enzymes relevant to SPM production are still able to produce SPMs including aspirin-triggered lipoxins, resolvins and protectins.^15^ A randomized trial in healthy humans showed that aspirin treatment for 8 weeks was able to increase plasma levels of 15-epi-LXA_4_^43^; this increase in aspirin-triggered SPMs has been supported by studies in others matrices and populations (e.g. skin inflammation^27^ or tuberculous meningitis^44^). In our study, participants could take aspirin at any time during the day, so one possible explanation for the lack of increase observed in PWH is that the time range of aspirin intake might make it more difficult to detect increases in these aspirin-triggered SPMs at the time of sample collection. However, we also did not note any increases in these SPMs compared to baseline despite the participants taking aspirin for 12 weeks, with an alternate explanation being that PWH on suppressive ART may not produce increased levels of SPMs in response to aspirin. This is consistent with our earlier studies showing that there is an impaired aspirin response in PWH based on their effects on platelet activating pathways compared to SN.^29^^,^^30^ These results suggest that COX-2 might potentially be less inhibited by aspirin in PWH but will need to be confirmed along with further assessment of the mechanisms.

The limitations of our study are related to assessment of lipid mediators only at the beginning and end of treatment, merging samples from multiple studies (i.e. ACTG, MACS/WIHS) and limited data on gut integrity markers. The mechanisms behind the observed changes in lipid mediators of inflammation in PWH are not clear and future studies will need to address this. Despite these limitations, our study has multiple strengths. These include well-characterized and well-matched study populations, measurement of a comprehensive panel of pro- and anti-inflammatory lipid mediators along with paired soluble protein marker data, and a randomized controlled design to study the effect of aspirin treatment on lipid mediators.

In conclusion, we observed distinct lipid mediator profiles among PWH compared to SN individuals. There were also relationships observed between lipid and markers of monocyte activation important in CVD. n−3 DPA-derived RvD5, linked with improved intestinal integrity, were inversely associated with sCD14 in PWH, suggesting intervention potential for this SPMs to improve intestinal integrity and reduce monocyte activation. We also noted that aspirin did not increase levels of aspirin-triggered SPMs in PWH, in line with other findings showing a defective aspirin response in PWH, and could help partly explain why aspirin was unable to reduce monocyte activation and systemic inflammation in PWH.

## Data sharing statement

The authors confirm that all data are available upon request (sdac.data@sdac.harvard.edu for ACTG data and programming code; https://statepi.jhsph.edu/mwccs/work-with-us/ for MWCCS data) and approval by the relevant networks.

## Declaration of interests

PCT: Merck has provided her institution with funding for her research; Gilead and Lilly have also provided her institution with funding for her to conduct industry-sponsored clinical trials. PWH: Gilead has provided funding to his institution, and Merck has provided donation of study drug for NIH-sponsored trial. He has also received consulting fees and other support from Viiv Healthcare, Biotron, Gilead and Longeveron. JD is an inventor on patents related to the composition of matter and/or use of pro-resolving mediators some of which are licensed by Brigham and Women's Hospital or Queen Mary University of London for clinical development. AG: NIH, UNITAID and CDC have provided funding to her institution. TTB has received consulting fees from ViiV Healthcare, Theratechnologies, Janssen, Merck and Gilead. JAA: Atea, Emergent Biosolutions, Frontier Technologies, Gilead Sciences, GSK, Janssen, Merck, Pfizer, Regeneron and Viiv Healthcare have provided her institution with funding. RS: funding for current work was provided to institution by NIH and the ACTG network. The remaining authors have no conflicts of interest to declare.

## Figures and Tables

**Supplementary Fig S6:**
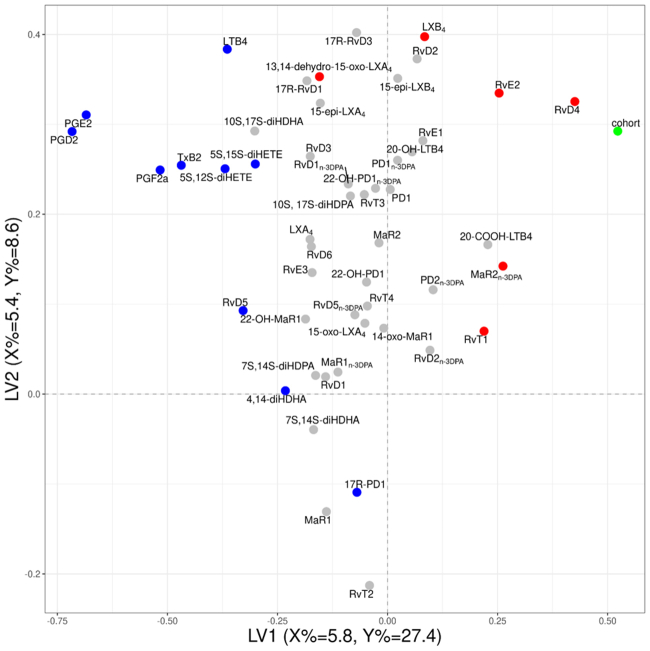


## Appendix A Supplementary data

Supplement_Combined
