# Supplementary material for: Pro-inflammatory and pro-resolving lipid mediators of inflammation in HIV: effect of aspirin intervention
Source: eBioMedicine. 2023 Feb 13;89:104468. doi: 10.1016/j.ebiom.2023.104468 (PMC10025757; doi:10.1016/j.ebiom.2023.104468)
Supplement: Supplement_Combined [file mmc1.docx]

**Supplementary File – Table of Contents**

**Tables of Content**

Supplementary Methods Pages 2-3

Supplementary Tables Pages 4-13

References Page 14

Supplementary Figure Legends Page 15

**Methods:**

**Study population:**

All PWH with available baseline plasma samples (N=110) from the AIDS Clinical Trials Group (ACTG) A5331 study (NCT02155985) were included in this analysis. The participants in A5331 were enrolled at 15 study sites in the United States from August 2014-March 2015. Exclusion criteria included use of immunosuppressive medications, frequent use of aspirin or aspirin products (i.e. an average of 2 or more times per week in the past 12 weeks), recent severe illness, liver or kidney disease, pregnant or breastfeeding, and uncontrolled diabetes^1^. The A5331 trial randomized 121 participants but the efficacy analyses were based on a per-protocol population consisting of 113 participants. Therefore, our sample size of n=110 represents a very high proportion of the A5331 analysis population.

Matched adult SN (87 men, 20 women) were selected from a large ongoing cohort study in the United States: The Multicenter AIDS Cohort Study (MACS)^2^ - Women’s Interagency HIV Study (WIHS)^3^ Combined Cohort Study^4^. After applying the eligibility criteria of the A5331 study, SN were matched 1:1 to PWH on sex, age, body mass index (BMI), race/ethnicity, smoking status, drinking status and statin use.

**Ethics approval**

Study participants provided written informed consent, including for future use of samples, for the parent studies (A5331, MACS and WIHS). The parent studies were approved by ethics committees in each study site. Our specific study on lipid mediators was also approved by Institutional Review Boards (IRBs) at Columbia University (AAAS0191), Johns Hopkins University (IRB00144189) and University of California San Francisco (194300). All methods were performed in accordance with the relevant guidelines and regulations.

**Laboratory Assessment:**

Plasma collection

EDTA plasma was collected from participants as part of each study. The plasma samples from PWH in A5331 (baseline/pre-intervention) and SN in MACS were matched based upon samples having been collected during the same time period. The samples from WIHS, however, were collected at a later time period specifically for this study as archived EDTA plasma was not available from the parent study during the same time period as when A5331 samples were collected. For the A5331 PWH, plasma samples after 12 weeks of aspirin intervention were also used for this study. Samples were stored at -80ºC until shipment for lipid mediator assessment at William Harvey Research Institute in the United Kingdom.

Soluble and cellular markers of inflammation

Plasma levels of soluble CD14 (sCD14), sCD163, interleukin-6 (IL-6) were measured at baseline as part of the parent A5331 study using enzyme-linked immunosorbent assays (R&D Systems, Minneapolis, MN) ^1^. The A5331 study also measured CD4 T-cell activation (CD38+HLA-DR+) using flow cytometry of peripheral blood mononuclear cells, as detailed elsewhere ^1^.

**Statistical analysis**:

A total of 50 unique lipid mediators were measured in plasma samples, with 42 SPMs and 8 pro-inflammatory lipid mediators (**Figure 1 and Supplementary Table 1**). These 50 lipid mediators were also categorized into 12 metabolome families (9 SPMs and 3 pro-inflammatory) (**Figure 1 and Supplementary Table 1**). Each of the 12 metabolome concentrations were calculated as the sum of the individual mediators within that metabolome. For all analyses, the values of the individual mediators and metabolomes were log_10_-transformed as the distribution were right-skewed; prior to this transformation, values of zero were imputed as one half of the smallest observed non-zero value within each mediator/metabolome.

A primary objective of this study was to compare plasma levels of i) lipid mediators and ii) metabolomes between PWH (pre-intervention) and SN. To visually examine how mediators/metabolomes and cohorts (PWH vs. SN) cluster together, hierarchical clustering heat maps were created for mediators/metabolomes (heat map columns) with cohort (heat map rows) using the Euclidean distance for clustering. Heat maps were also created as stacked bar plots for mediator/metabolome abundance, with abundance calculated as the proportion of individual mediators/metabolomes prior to log-transformation and without zero imputation.

Analysis of covariance (ANCOVA) was used to assess the HIV effect on the 12 metabolomes after adjustment for sex, race/ethnicity, age, BMI, smoking status, drinking status and statin use. To control the false discovery rate (FDR) of these 12 analyses, the Benjamini-Hochberg procedure was used and the FDR-adjusted p-values were assessed at the 5% significance level.

For the individual mediators, we used a data reduction method to account for the large numbers of mediators. As we wanted to account for the HIV status of each participant, the supervised partial least squares discriminant analysis (PLS-DA) method was used. PLS-DA reduces the dimension of the mediator data (predictors) while also maximizing the covariance between this reduced predictor data and HIV status (outcome). Two latent variables were extracted that explained 27% (i.e. substantial proportion of variation) and 9% of outcome variation. Variable importance for the projection (VIP) values >1 were used to identify predictors of “above average importance” in the fitting model for both predictors and outcomes. Sixteen mediators and pathway markers had VIP>1, where 6 were positively associated and 10 were negatively associated with HIV+ status (**Supplementary Figure 6)**. These 16 identified mediators were each used in exploratory ANCOVA models with unadjusted p-values assessed at the 1% significance level.

The second objective of this study was to determine the association of lipid mediators of inflammation with soluble (protein) and cellular markers of immune activation among PWH (i.e. A5331 study participants at baseline). The primary outcome variable in this analysis was “high” inflammation defined as the highest quartile (Q4) compared to Q1-Q3 of the monocyte activation marker sCD14. Other exploratory outcome variables were “high inflammation” for sCD163, IL-6 and CD4 T-cell activation (CD38+HLA-DR+). Logistic regression was used to determine the association of high inflammation (outcome variable) with i) individual metabolome, ii) individual principal components (derived from unsupervised principal components analysis) of the metabolome, and iii) individual principal components of the mediators. The association of individual metabolomes with “high” sCD14 used FDR-adjusted p-values were assessed at the 5% significance level. All other exploratory analyses used unadjusted p-values assessed at the 1% significance level.

The final objective of this study was to study whether and how the administration of aspirin changed levels of lipid mediators among PWH (i.e. A5331 participants before and after the intervention). We conducted analysis of variance (ANOVA) to assess the effect of each aspirin arm in A5331 (100 mg and 300 mg arms) relative to placebo on 12-week change (i.e. change in lipid mediators/metabolomes from baseline to 12 weeks) in mediators and metabolomes. If a statistically significant difference was observed between either the 100 mg or 300 mg arm compared to placebo, the difference between the two aspirin arms were also estimated using ANOVA. The model estimates are presented as mean fold change within each arm, and mean fold change % difference between arms. All analyses used unadjusted p-values assessed at the 1% significance level.

The statistical analyses and visualizations were generated using SAS software (Version 9.4 for Linux. Copyright © 2016 SAS Institute Inc., Cary, NC, USA) and R software (Version 3.6.0 for Linux. Copyright © 2019 R Foundation for Statistical Computing, Vienna, Austria).

**Supplementary Tables**

**Supplementary Table 1**: Lipid mediators from lipid mediator families

| **Precursor Molecule** | **Metabolome** | **SPMs** | **Classic Eicosanoids** |
| --- | --- | --- | --- |
| Docosahexaenoic acid (DHA) | Resolvins D-series (DHA RvD) | RvD1; RvD2; RvD3; RvD4; RvD5; RvD6; 17R-RvD1; 17R-RvD3 |  |
|  | Protectins (DHA PD) | PD1; 10S,17S-diHDHA; 22-OH-PD1; 17R-PD1 |  |
|  | Maresins (DHA MaR) | MaR1; 7S,14S-diHDHA; MaR2; 22-OH-MaR1; 14-oxo-MaR1; 4,14-diHDHA |  |
| N-3 docosapentaenoic acid (n-3 DPA) | Resolvins 13-series (n-3 DPA RvT) | RvT1; RvT2; RvT3; RvT4 |  |
|  | Resolvins D-series (n-3 DPA RvD) | RvD1­_n-3 DPA_; RvD2_n-3 DPA_; RvD5_n-3 DPA_ |  |
|  | Protectins (n-3 DPA PD) | PD1_n-3 DPA_; PD2_n-3 DPA_; 10S,17S-diHDPA; 22-OH-PD1_n-3 DPA_ |  |
|  | Maresins (n-3 DPA MaR) | MaR1_n-3 DPA_; MaR2_n-3 DPA_; 17S,14S-diHDPA |  |
| Eicosapentanoic acid (EPA) | Resolvins E-series (EPA RvE) | RvE1; RvE2; RvE3 |  |
| Arachidonic acid (AA) | Lipoxins (AA LX) | LXA_4_; LXB_4_; 5S,15S-diHETE; 13,14-dehydro-15-oxo-LXA_4_; 15-oxo-LXA_4_; 15-epi-LXA_4_; 15-epi-LXB_4_ |  |
|  | Leukotrienes (AA LT) |  | LTB_4_; 5S,12S-diHETE; 20-OH-LTB_4_; 20-COOH-LTB_4_ |
|  | Prostaglandins (AA PG) |  | PGE_2_; PGD_2_; PGF_2a_ |
|  | Thromboxane (AA Tx) |  | TxB­_2_ |

**Supplementary Table 1 Legend**: 50 unique lipid mediators (8 classic eicosanoids and 42 specialized pro-resolving mediators (SPMs) from these distinct mediator families that were measured in this study.

**Supplementary Table 2: Levels of Lipid mediator metabolome**

|  | | **Cohort** | |  |
| --- | --- | --- | --- | --- |
| **Metabolome** |  | **PWH (N=110)** | **SN (N=107)** | **Total (N=217)** |
| DHA - RvD (log_10_ pg/mL) | Median (Q1, Q3) | 0.23 (-0.08, 0.62) | 0.00 (-0.37, 0.36) | 0.12 (-0.33, 0.47) |
|  | | | | |
| DHA - PD (log_10_ pg/mL) | Median (Q1, Q3) | -0.15 (-0.66, 0.14) | -0.17 (-0.55, 0.09) | -0.15 (-0.57, 0.11) |
|  | | | | |
| DHA - MaR (log_10_ pg/mL) | Median (Q1, Q3) | 0.78 (-0.19, 1.20) | 0.84 (0.21, 1.32) | 0.83 (0.05, 1.26) |
|  | | | | |
| n-3 DPA - RvT (log_10_ pg/mL) | Median (Q1, Q3) | 0.30 (-0.19, 0.62) | 0.32 (-0.10, 0.61) | 0.31 (-0.12, 0.61) |
|  | | | | |
| n-3 DPA - RvD_n-3 DPA_ (log_10_ pg/mL) | Median (Q1, Q3) | -0.31 (-1.35, 0.41) | -0.11 (-0.70, 0.26) | -0.24 (-0.85, 0.31) |
|  | | | | |
| n-3 DPA - PD_n-3 DPA_ (log_10_ pg/mL) | Median (Q1, Q3) | -0.17 (-1.22, 0.25) | -0.35 (-1.22, 0.06) | -0.30 (-1.22, 0.18) |
|  | | | | |
| n-3 DPA - MaR_n-3 DPA_ (log_10_ pg/mL) | Median (Q1, Q3) | 0.30 (0.05, 0.66) | 0.28 (0.03, 0.53) | 0.29 (0.04, 0.56) |
|  | | | | |
| EPA - RvE (log_10_ pg/mL) | Median (Q1, Q3) | -0.66 (-1.46, 0.15) | -0.64 (-1.46, 0.23) | -0.66 (-1.46, 0.21) |
|  | | | | |
| AA - LX (log_10_ pg/mL) | Median (Q1, Q3) | 1.18 (0.72, 1.52) | 1.02 (0.56, 1.57) | 1.11 (0.68, 1.52) |
|  | | | | |
| AA - LT (log_10_ pg/mL) | Median (Q1, Q3) | -0.03 (-0.68, 0.46) | -0.17 (-0.68, 0.46) | -0.12 (-0.68, 0.46) |
|  | | | | |
| AA - PG (log_10_ pg/mL) | Median (Q1, Q3) | 0.65 (0.40, 0.94) | 0.99 (0.66, 1.25) | 0.79 (0.50, 1.10) |
|  | | | | |
| AA - Tx (log_10_ pg/mL) | Median (Q1, Q3) | 0.75 (0.17, 1.31) | 1.03 (0.56, 1.55) | 0.92 (0.27, 1.47) |
|  | | | | |

Supplementary Table 2 Legend: Log_10_ median levels and interquartile range of metabolomes of lipid mediators are shown for the overall population and by HIV status

**Supplementary Table 3: Levels of Lipid mediator**

|  | | **Cohort** | |  |
| --- | --- | --- | --- | --- |
| **Metabolite** |  | **PWH (N=110)** | **SN (N=107)** | **Total (N=217)** |
| RvD1 (log_10_ pg/mL) | Median (Q1, Q3) | -1.05 (-1.05, -0.41) | -1.05 (-1.05, -0.32) | -1.05 (-1.05, -0.41) |
|  | | | | |
| RvD2 (log_10_ pg/mL) | Median (Q1, Q3) | -0.89 (-0.89, -0.89) | -0.89 (-0.89, -0.89) | -0.89 (-0.89, -0.89) |
|  | | | | |
| RvD3 (log_10_ pg/mL) | Median (Q1, Q3) | -1.40 (-1.40, -1.40) | -1.40 (-1.40, -1.40) | -1.40 (-1.40, -1.40) |
|  | | | | |
| RvD4 (log_10_ pg/mL) | Median (Q1, Q3) | -0.69 (-0.69, 0.12) | -0.69 (-0.69, -0.69) | -0.69 (-0.69, -0.08) |
|  | | | | |
| RvD5 (log_10_ pg/mL) | Median (Q1, Q3) | -1.26 (-1.26, -0.36) | -0.60 (-1.26, -0.31) | -0.92 (-1.26, -0.36) |
|  | | | | |
| RvD6 (log_10_ pg/mL) | Median (Q1, Q3) | -1.10 (-1.10, -1.10) | -1.10 (-1.10, -0.51) | -1.10 (-1.10, -0.64) |
|  | | | | |
| 17R-RvD1 (log_10_ pg/mL) | Median (Q1, Q3) | -1.05 (-1.05, -1.05) | -1.05 (-1.05, -0.44) | -1.05 (-1.05, -0.51) |
|  | | | | |
| 17R-RvD3 (log_10_ pg/mL) | Median (Q1, Q3) | -1.46 (-1.46, -1.46) | -1.46 (-1.46, -1.46) | -1.46 (-1.46, -1.46) |
|  | | | | |
| PD1 (log_10_ pg/mL) | Median (Q1, Q3) | -0.57 (-1.19, -0.11) | -0.54 (-1.19, -0.25) | -0.55 (-1.19, -0.21) |
|  | | | | |
| 10S,17S-diHDHA (log_10_ pg/mL) | Median (Q1, Q3) | -1.26 (-1.26, -0.46) | -1.26 (-1.26, -0.41) | -1.26 (-1.26, -0.44) |
|  | | | | |
| 22-OH-PD1 (log_10_ pg/mL) | Median (Q1, Q3) | -0.96 (-0.96, -0.96) | -0.96 (-0.96, -0.96) | -0.96 (-0.96, -0.96) |
|  | | | | |
| 17R-PD1 (log_10_ pg/mL) | Median (Q1, Q3) | -1.30 (-1.30, -1.30) | -1.30 (-1.30, -0.60) | -1.30 (-1.30, -0.70) |
|  | | | | |
| MaR1 (log_10_ pg/mL) | Median (Q1, Q3) | -0.36 (-0.36, 0.68) | 0.21 (-0.36, 0.90) | 0.08 (-0.36, 0.77) |
|  | | | | |
| 7S,14S-diHDHA (log_10_ pg/mL) | Median (Q1, Q3) | -0.43 (-0.43, 0.55) | -0.43 (-0.43, 0.59) | -0.43 (-0.43, 0.56) |
|  | | | | |
| MaR2 (log_10_ pg/mL) | Median (Q1, Q3) | -0.55 (-0.55, -0.55) | -0.55 (-0.55, -0.55) | -0.55 (-0.55, -0.55) |
|  | | | | |
| 22-OH-MaR1 (log_10_ pg/mL) | Median (Q1, Q3) | -0.44 (-0.44, 0.03) | -0.44 (-0.44, 0.49) | -0.44 (-0.44, 0.13) |
|  | | | | |
| 14-oxo-MaR1 (log_10_ pg/mL) | Median (Q1, Q3) | -0.80 (-0.80, -0.80) | -0.80 (-0.80, -0.80) | -0.80 (-0.80, -0.80) |
|  | | | | |
| 4,14-diHDHA (log_10_ pg/mL) | Median (Q1, Q3) | -0.96 (-0.96, -0.96) | -0.96 (-0.96, -0.96) | -0.96 (-0.96, -0.96) |
|  | | | | |
| RvT1 (log_10_ pg/mL) | Median (Q1, Q3) | -1.30 (-1.30, -0.47) | -1.30 (-1.30, -0.66) | -1.30 (-1.30, -0.55) |
|  | | | | |
| RvT2 (log_10_ pg/mL) | Median (Q1, Q3) | -0.84 (-1.35, -0.28) | -0.70 (-1.35, -0.21) | -0.80 (-1.35, -0.22) |
|  | | | | |
| RvT3 (log_10_ pg/mL) | Median (Q1, Q3) | -1.35 (-1.35, -1.35) | -1.35 (-1.35, -1.35) | -1.35 (-1.35, -1.35) |
|  | | | | |
| RvT4 (log_10_ pg/mL) | Median (Q1, Q3) | -0.01 (-1.07, 0.42) | 0.02 (-0.43, 0.42) | 0.00 (-0.55, 0.42) |
|  | | | | |
| RvD1_n-3 DPA_ (log_10_ pg/mL) | Median (Q1, Q3) | -1.35 (-1.35, -0.74) | -1.35 (-1.35, -0.72) | -1.35 (-1.35, -0.72) |
|  | | | | |
| RvD2_n-3 DPA_ (log_10_ pg/mL) | Median (Q1, Q3) | -1.30 (-1.30, -0.19) | -0.80 (-1.30, -0.39) | -0.92 (-1.30, -0.35) |
|  | | | | |
| RvD5_n-3 DPA_ (log_10_ pg/mL) | Median (Q1, Q3) | -0.98 (-0.98, -0.10) | -0.98 (-0.98, -0.05) | -0.98 (-0.98, -0.08) |
|  | | | | |
| PD1_n-3 DPA_ (log_10_ pg/mL) | Median (Q1, Q3) | -1.19 (-1.19, -0.80) | -1.19 (-1.19, -0.85) | -1.19 (-1.19, -0.85) |
|  | | | | |
| PD2_n-3 DPA_ (log_10_ pg/mL) | Median (Q1, Q3) | -1.22 (-1.22, -1.22) | -1.22 (-1.22, -1.22) | -1.22 (-1.22, -1.22) |
|  | | | | |
| 10S, 17S-diHDPA (log_10_ pg/mL) | Median (Q1, Q3) | -1.30 (-1.30, -0.70) | -1.30 (-1.30, -0.64) | -1.30 (-1.30, -0.68) |
|  | | | | |
| 22-OH-PD1_n-3 DPA_ (log_10_ pg/mL) | Median (Q1, Q3) | -1.15 (-1.15, 0.05) | -1.15 (-1.15, -0.28) | -1.15 (-1.15, -0.11) |
|  | | | | |
| MaR1_n-3 DPA_ (log_10_ pg/mL) | Median (Q1, Q3) | -0.89 (-0.89, -0.89) | -0.89 (-0.89, -0.33) | -0.89 (-0.89, -0.41) |
|  | | | | |
| MaR2_n-3 DPA_ (log_10_ pg/mL) | Median (Q1, Q3) | 0.16 (-0.12, 0.61) | 0.12 (-0.21, 0.45) | 0.14 (-0.14, 0.47) |
|  | | | | |
| 7S,14S-diHDPA (log_10_ pg/mL) | Median (Q1, Q3) | -0.85 (-0.85, -0.85) | -0.85 (-0.85, -0.85) | -0.85 (-0.85, -0.85) |
|  | | | | |
| RvE1 (log_10_ pg/mL) | Median (Q1, Q3) | -1.10 (-1.10, -1.10) | -1.10 (-1.10, -1.10) | -1.10 (-1.10, -1.10) |
|  | | | | |
| RvE2 (log_10_ pg/mL) | Median (Q1, Q3) | -0.29 (-0.29, -0.29) | -0.29 (-0.29, -0.29) | -0.29 (-0.29, -0.29) |
|  | | | | |
| RvE3 (log_10_ pg/mL) | Median (Q1, Q3) | -1.46 (-1.46, -0.15) | -1.46 (-1.46, 0.13) | -1.46 (-1.46, -0.09) |
|  | | | | |
| LXA_4_ (log_10_ pg/mL) | Median (Q1, Q3) | -1.46 (-1.46, -1.46) | -1.46 (-1.46, -0.92) | -1.46 (-1.46, -1.10) |
|  | | | | |
| LXB_4_ (log_10_ pg/mL) | Median (Q1, Q3) | 0.88 (-0.15, 1.35) | 0.43 (-0.15, 1.28) | 0.60 (-0.15, 1.33) |
|  | | | | |
| 5S,15S-diHETE (log_10_ pg/mL) | Median (Q1, Q3) | -1.22 (-1.82, -0.27) | -0.70 (-1.82, -0.13) | -1.00 (-1.82, -0.21) |
|  | | | | |
| 13,1_4_-dehydro-15-oxo-LXA_4_ (log_10_ pg/mL) | Median (Q1, Q3) | -1.35 (-1.35, -0.42) | -1.35 (-1.35, -0.92) | -1.35 (-1.35, -0.70) |
|  | | | | |
| 15-oxo-LXA_4_ (log_10_ pg/mL) | Median (Q1, Q3) | -0.41 (-1.30, 0.21) | -0.54 (-1.30, 0.41) | -0.48 (-1.30, 0.22) |
|  | | | | |
| 15-epi-LXA_4_ (log_10_ pg/mL) | Median (Q1, Q3) | 0.19 (-0.24, 0.64) | 0.17 (-0.31, 0.75) | 0.17 (-0.28, 0.69) |
|  | | | | |
| 15-epi-LXB_4_ (log_10_ pg/mL) | Median (Q1, Q3) | -0.57 (-0.72, 0.45) | -0.72 (-0.72, 0.17) | -0.72 (-0.72, 0.37) |
|  | | | | |
| LTB_4_ (log_10_ pg/mL) | Median (Q1, Q3) | -0.44 (-1.22, 0.06) | -0.42 (-0.85, -0.01) | -0.42 (-1.00, 0.00) |
|  | | | | |
| 5S,12S-diHETE (log_10_ pg/mL) | Median (Q1, Q3) | -1.70 (-1.70, -0.66) | -1.30 (-1.70, -0.48) | -1.70 (-1.70, -0.54) |
|  | | | | |
| 20-OH-LTB_4_ (log_10_ pg/mL) | Median (Q1, Q3) | -1.15 (-1.15, -1.15) | -1.15 (-1.15, -1.15) | -1.15 (-1.15, -1.15) |
|  | | | | |
| 20-COOH-LTB_4_ (log_10_ pg/mL) | Median (Q1, Q3) | -1.10 (-1.10, -1.10) | -1.10 (-1.10, -1.10) | -1.10 (-1.10, -1.10) |
|  | | | | |
| PGE_2_ (log_10_ pg/mL) | Median (Q1, Q3) | 0.22 (-0.10, 0.50) | 0.66 (0.10, 0.99) | 0.35 (-0.03, 0.81) |
|  | | | | |
| PGD_2_ (log_10_ pg/mL) | Median (Q1, Q3) | 0.07 (-0.22, 0.36) | 0.37 (0.09, 0.75) | 0.21 (-0.07, 0.57) |
|  | | | | |
| PGF_2a_ (log_10_ pg/mL) | Median (Q1, Q3) | -0.04 (-0.47, 0.32) | 0.23 (-0.15, 0.58) | 0.09 (-0.29, 0.45) |
|  | | | | |
| TxB_2_ (log_10_ pg/mL) | Median (Q1, Q3) | 0.75 (0.17, 1.31) | 1.03 (0.56, 1.55) | 0.92 (0.27, 1.47) |
|  | | | | |

Supplementary Table 3 Legend: Median levels and interquartile range of lipid mediators are shown for the overall population and by HIV status

**Supplementary Table 4: Differences in lipid mediators by HIV status**

| **Metabolite Outcome** | **Observations Used** | **PWH Geometric LS Mean (95% CI)** | **SN Geometric LS Mean (95% CI)** | **Fold Difference (95% CI)** | **Raw p-value** |
| --- | --- | --- | --- | --- | --- |
| RvD4 (pg/mL) | 212 | 0.57 (0.46, 0.72) | 0.28 (0.22, 0.35) | 2.04 (1.47, 2.82) | **<0.001** |
| RvD5 (pg/mL) | 212 | 0.14 (0.11, 0.18) | 0.20 (0.16, 0.26) | 0.69 (0.49, 0.95) | 0.024 |
| 17R-PD1 (pg/mL) | 212 | 0.08 (0.06, 0.09) | 0.10 (0.08, 0.12) | 0.79 (0.62, 1.02) | 0.067 |
| 4,14-diHDHA (pg/mL) | 212 | 0.12 (0.11, 0.14) | 0.16 (0.14, 0.18) | 0.77 (0.64, 0.93) | **0.006** |
| RvT1 (pg/mL) | 212 | 0.13 (0.11, 0.17) | 0.09 (0.07, 0.11) | 1.51 (1.10, 2.07) | 0.011 |
| MaR2_n-3 DPA_ (pg/mL) | 212 | 1.92 (1.51, 2.45) | 1.09 (0.86, 1.39) | 1.76 (1.25, 2.49) | **0.001** |
| RvE2 (pg/mL) | 212 | 0.59 (0.55, 0.63) | 0.52 (0.49, 0.55) | 1.14 (1.03, 1.25) | **0.008** |
| LXB_4_ (pg/mL) | 212 | 6.32 (4.34, 9.22) | 3.65 (2.50, 5.33) | 1.73 (1.01, 2.96) | 0.045 |
| 5S,15S-diHETE (pg/mL) | 212 | 0.09 (0.06, 0.13) | 0.15 (0.11, 0.22) | 0.60 (0.36, 1.01) | 0.054 |
| 13,14-dehydro-15-oxo-LXA_4_ (pg/mL) | 212 | 0.13 (0.09, 0.17) | 0.10 (0.07, 0.14) | 1.23 (0.79, 1.93) | 0.36 |
| LTB_4_ (pg/mL) | 212 | 0.26 (0.18, 0.37) | 0.43 (0.30, 0.63) | 0.59 (0.35, 1.01) | 0.052 |
| 5S,12S-diHETE (pg/mL) | 212 | 0.06 (0.05, 0.09) | 0.10 (0.07, 0.13) | 0.65 (0.41, 1.04) | 0.075 |
| PGE_2_ (pg/mL) | 212 | 1.52 (1.13, 2.05) | 3.36 (2.50, 4.52) | 0.45 (0.30, 0.69) | **<0.001** |
| PGD_2_ (pg/mL) | 212 | 1.12 (0.88, 1.44) | 2.61 (2.04, 3.34) | 0.43 (0.30, 0.61) | **<0.001** |
| PGF_2a_ (pg/mL) | 212 | 0.79 (0.61, 1.02) | 1.64 (1.27, 2.13) | 0.48 (0.33, 0.69) | **<0.001** |
| TxB_2_ (pg/mL) | 212 | 4.18 (2.75, 6.34) | 9.67 (6.37, 14.67) | 0.43 (0.24, 0.78) | **0.006** |

Supplementary Table 4 Legend**:** Analysis of covariance was conducted to assess the differences in lipid mediators by HIV status. Only 16 lipid mediators with variable importance in projection (VIP)>1 from partial least squares-discriminant analysis are shown above. All models are adjusted for sex, race/ethnicity, age, body mass index, smoking, drinking and statin use.

**Supplementary Table 5: Relationship of lipid mediator metabolomes with high sCD14 among PWH**

| **Metabolome** | **Observations Used** | **Odds Ratio (95% CI)** | **Raw p-value** | **FDR-adjusted p-value** |
| --- | --- | --- | --- | --- |
| DHA - RvD | 110 | 2.03 (0.98, 4.19) | 0.056 | 0.17 |
| DHA - PD | 110 | 1.86 (0.92, 3.78) | 0.085 | 0.17 |
| DHA - MaR | 110 | 0.73 (0.44, 1.21) | 0.23 | 0.27 |
| n-3 DPA - RvT | 110 | 1.90 (0.91, 3.98) | 0.087 | 0.17 |
| n-3 DPA - RvD_n-3 DPA_ | 110 | 2.14 (1.20, 3.82) | 0.010 | 0.12 |
| n-3 DPA - PD_n-3 DPA_ | 110 | 1.27 (0.66, 2.43) | 0.48 | 0.48 |
| n-3 DPA - MaR_n-3 DPA_ | 110 | 1.62 (0.71, 3.71) | 0.26 | 0.28 |
| EPA - RvE | 110 | 1.42 (0.88, 2.29) | 0.15 | 0.25 |
| AA - LX | 110 | 1.50 (0.80, 2.82) | 0.21 | 0.27 |
| AA - LT | 110 | 1.42 (0.82, 2.45) | 0.21 | 0.27 |
| AA - PG | 110 | 2.39 (0.99, 5.74) | 0.052 | 0.17 |
| AA - Tx | 110 | 1.68 (1.03, 2.73) | 0.038 | 0.17 |

Supplementary Table 5: Logistic regression analysis was used to assess the odds ratio (95% confidence intervals) of high sCD14 (defined as the highest quartile) for a unit increase in each of the metabolomes. Results are shown for raw adjusted p-values and false discovery rate (FDR) adjusted p-values.

**Supplementary Table 6:**  **Effect of aspirin on individual lipid mediators among PWH**

| **Metabolite** | **Treatment Arm** | **Mean fold Δ (95% CI)** | **Mean fold Δ % difference (95% CI) vs. Placebo** | **Raw p-value vs. Placebo** | **Mean fold Δ % difference (95% CI) vs. 100 mg** | **Raw p-value vs. 100 mg** |
| --- | --- | --- | --- | --- | --- | --- |
| RvD1 | 300 mg | 0.93 (0.61, 1.41) | -5.6 (-47.8, 70.8) | 0.85 | - | - |
|  | 100 mg | 0.93 (0.62, 1.41) | -5.5 (-47.8, 71.0) | 0.85 |  |  |
|  | Placebo | 0.99 (0.65, 1.51) |  |  |  |  |
| RvD2 | 300 mg | 0.90 (0.67, 1.22) | -15.5 (-44.7, 29.1) | 0.43 | - | - |
|  | 100 mg | 0.82 (0.61, 1.10) | -23.7 (-50.0, 16.6) | 0.21 |  |  |
|  | Placebo | 1.07 (0.79, 1.44) |  |  |  |  |
| RvD3 | 300 mg | 1.20 (0.84, 1.72) | 21.7 (-27.3, 103.7) | 0.45 | - | - |
|  | 100 mg | 0.97 (0.68, 1.40) | -1.4 (-41.1, 65.1) | 0.96 |  |  |
|  | Placebo | 0.99 (0.68, 1.42) |  |  |  |  |
| RvD4 | 300 mg | 0.60 (0.38, 0.93) | -8.1 (-51.1, 72.6) | 0.79 | - | - |
|  | 100 mg | 0.46 (0.30, 0.72) | -28.8 (-62.1, 33.8) | 0.29 |  |  |
|  | Placebo | 0.65 (0.42, 1.02) |  |  |  |  |
| RvD5 | 300 mg | 1.40 (0.83, 2.36) | 40.2 (-33.5, 195.6) | 0.37 | - | - |
|  | 100 mg | 0.86 (0.51, 1.45) | -13.8 (-59.1, 81.7) | 0.69 |  |  |
|  | Placebo | 1.00 (0.59, 1.70) |  |  |  |  |
| RvD6 | 300 mg | 1.16 (0.75, 1.81) | -38.8 (-67.3, 14.6) | 0.12 | - | - |
|  | 100 mg | 1.09 (0.70, 1.69) | -42.7 (-69.4, 7.4) | 0.082 |  |  |
|  | Placebo | 1.90 (1.22, 2.97) |  |  |  |  |
| 17R-RvD1 | 300 mg | 1.05 (0.72, 1.53) | 17.2 (-31.8, 101.4) | 0.56 | - | - |
|  | 100 mg | 0.78 (0.53, 1.14) | -12.9 (-49.3, 49.6) | 0.61 |  |  |
|  | Placebo | 0.90 (0.61, 1.32) |  |  |  |  |
| 17R-RvD3 | 300 mg | 0.85 (0.61, 1.19) | -0.6 (-38.1, 59.7) | 0.98 | - | - |
|  | 100 mg | 0.79 (0.57, 1.11) | -7.4 (-42.4, 48.8) | 0.75 |  |  |
|  | Placebo | 0.86 (0.61, 1.20) |  |  |  |  |
| PD1 | 300 mg | 1.40 (0.84, 2.33) | 38.7 (-32.7, 185.6) | 0.37 | - | - |
|  | 100 mg | 0.66 (0.40, 1.10) | -34.6 (-68.3, 34.7) | 0.25 |  |  |
|  | Placebo | 1.01 (0.60, 1.69) |  |  |  |  |
| 10S,17S-diHDHA | 300 mg | 0.92 (0.53, 1.60) | 48.3 (-32.0, 223.2) | 0.32 | - | - |
|  | 100 mg | 1.02 (0.59, 1.76) | 63.1 (-25.2, 255.5) | 0.22 |  |  |
|  | Placebo | 0.62 (0.36, 1.09) |  |  |  |  |
| 22-OH-PD1 | 300 mg | 0.89 (0.61, 1.28) | -9.5 (-46.3, 52.7) | 0.71 | - | - |
|  | 100 mg | 0.89 (0.62, 1.29) | -8.9 (-46.0, 53.6) | 0.72 |  |  |
|  | Placebo | 0.98 (0.68, 1.42) |  |  |  |  |
| 17R-PD1 | 300 mg | 0.98 (0.67, 1.42) | 27.2 (-25.2, 116.3) | 0.37 | - | - |
|  | 100 mg | 0.94 (0.65, 1.36) | 22.0 (-28.2, 107.4) | 0.46 |  |  |
|  | Placebo | 0.77 (0.53, 1.12) |  |  |  |  |
| MaR1 | 300 mg | 0.71 (0.36, 1.37) | -1.1 (-61.6, 154.6) | 0.98 | - | - |
|  | 100 mg | 1.26 (0.65, 2.44) | 75.7 (-31.8, 352.3) | 0.24 |  |  |
|  | Placebo | 0.72 (0.36, 1.40) |  |  |  |  |
| 7S,14S-diHDHA | 300 mg | 0.77 (0.42, 1.43) | -4.4 (-60.3, 130.2) | 0.92 | - | - |
|  | 100 mg | 1.02 (0.55, 1.90) | 26.9 (-47.3, 205.5) | 0.59 |  |  |
|  | Placebo | 0.81 (0.43, 1.51) |  |  |  |  |
| MaR2 | 300 mg | 1.19 (0.87, 1.65) | 32.3 (-16.3, 109.2) | 0.23 | - | - |
|  | 100 mg | 1.27 (0.92, 1.75) | 40.5 (-11.1, 122.1) | 0.14 |  |  |
|  | Placebo | 0.90 (0.65, 1.25) |  |  |  |  |
| 22-OH-MaR1 | 300 mg | 0.86 (0.53, 1.39) | -6.7 (-52.9, 84.8) | 0.84 | - | - |
|  | 100 mg | 1.14 (0.71, 1.85) | 24.3 (-37.2, 146.1) | 0.53 |  |  |
|  | Placebo | 0.92 (0.57, 1.50) |  |  |  |  |
| 14-oxo-MaR1 | 300 mg | 1.15 (0.78, 1.71) | -19.3 (-54.0, 41.6) | 0.45 | - | - |
|  | 100 mg | 0.93 (0.62, 1.37) | -35.1 (-63.0, 13.8) | 0.13 |  |  |
|  | Placebo | 1.43 (0.96, 2.13) |  |  |  |  |
| 4,14-diHDHA | 300 mg | 0.92 (0.70, 1.20) | -4.4 (-34.7, 39.8) | 0.81 | - | - |
|  | 100 mg | 1.19 (0.91, 1.56) | 24.2 (-15.1, 81.6) | 0.26 |  |  |
|  | Placebo | 0.96 (0.73, 1.26) |  |  |  |  |
| RvT1 | 300 mg | 0.72 (0.43, 1.20) | -30.2 (-66.5, 45.5) | 0.33 | - | - |
|  | 100 mg | 0.74 (0.44, 1.25) | -27.3 (-65.1, 51.6) | 0.39 |  |  |
|  | Placebo | 1.02 (0.61, 1.73) |  |  |  |  |
| RvT2 | 300 mg | 0.91 (0.53, 1.55) | 23.6 (-42.3, 164.7) | 0.58 | - | - |
|  | 100 mg | 1.10 (0.64, 1.88) | 50.0 (-30.0, 221.1) | 0.29 |  |  |
|  | Placebo | 0.73 (0.43, 1.26) |  |  |  |  |
| RvT3 | 300 mg | 0.81 (0.64, 1.02) | -30.9 (-50.4, -3.8) | 0.029 | - | - |
|  | 100 mg | 0.95 (0.75, 1.20) | -18.7 (-41.6, 13.2) | 0.22 |  |  |
|  | Placebo | 1.17 (0.92, 1.48) |  |  |  |  |
| RvT4 | 300 mg | 0.97 (0.49, 1.95) | 35.1 (-49.5, 261.6) | 0.55 | - | - |
|  | 100 mg | 0.85 (0.43, 1.70) | 18.1 (-55.9, 216.1) | 0.74 |  |  |
|  | Placebo | 0.72 (0.36, 1.45) |  |  |  |  |
| RvD1_n-3 DPA_ | 300 mg | 1.15 (0.75, 1.74) | -10.8 (-50.8, 61.5) | 0.70 | - | - |
|  | 100 mg | 1.29 (0.85, 1.96) | 0.4 (-44.6, 82.0) | 0.99 |  |  |
|  | Placebo | 1.29 (0.84, 1.96) |  |  |  |  |
| RvD2_n-3 DPA_ | 300 mg | 0.91 (0.46, 1.81) | 16.6 (-56.2, 209.8) | 0.76 | - | - |
|  | 100 mg | 1.10 (0.55, 2.19) | 40.8 (-47.0, 274.3) | 0.49 |  |  |
|  | Placebo | 0.78 (0.39, 1.57) |  |  |  |  |
| RvD5_n-3 DPA_ | 300 mg | 1.13 (0.67, 1.90) | 16.7 (-44.6, 145.7) | 0.68 | - | - |
|  | 100 mg | 1.36 (0.81, 2.30) | 41.0 (-33.1, 196.9) | 0.36 |  |  |
|  | Placebo | 0.97 (0.57, 1.64) |  |  |  |  |
| PD1_n-3 DPA_ | 300 mg | 1.00 (0.65, 1.53) | -15.4 (-54.0, 55.5) | 0.59 | - | - |
|  | 100 mg | 0.87 (0.57, 1.34) | -25.9 (-59.7, 36.2) | 0.33 |  |  |
|  | Placebo | 1.18 (0.76, 1.82) |  |  |  |  |
| PD2_n-3 DPA_ | 300 mg | 1.16 (0.91, 1.48) | 26.9 (-9.9, 78.7) | 0.17 | - | - |
|  | 100 mg | 0.83 (0.65, 1.05) | -9.8 (-35.9, 27.1) | 0.55 |  |  |
|  | Placebo | 0.92 (0.72, 1.17) |  |  |  |  |
| 10S, 17S-diHDPA | 300 mg | 0.86 (0.54, 1.36) | -43.3 (-70.7, 9.7) | 0.091 | - | - |
|  | 100 mg | 1.14 (0.72, 1.81) | -24.7 (-61.0, 45.6) | 0.40 |  |  |
|  | Placebo | 1.51 (0.95, 2.42) |  |  |  |  |
| 22-OH-PD1_n-3 DPA_ | 300 mg | 0.91 (0.59, 1.40) | -20.7 (-57.2, 47.2) | 0.46 | - | - |
|  | 100 mg | 0.82 (0.53, 1.27) | -28.3 (-61.3, 33.1) | 0.29 |  |  |
|  | Placebo | 1.15 (0.74, 1.78) |  |  |  |  |
| MaR1_n-3 DPA_ | 300 mg | 1.10 (0.73, 1.67) | -3.2 (-46.6, 75.4) | 0.91 | - | - |
|  | 100 mg | 0.99 (0.65, 1.51) | -12.8 (-51.9, 58.1) | 0.65 |  |  |
|  | Placebo | 1.14 (0.75, 1.74) |  |  |  |  |
| MaR2_n-3 DPA_ | 300 mg | 1.32 (0.84, 2.05) | 8.2 (-42.4, 103.3) | 0.81 | 152.0 (34.7, 371.5) | **0.004** |
|  | 100 mg | 0.52 (0.34, 0.81) | -57.1 (-77.2, -19.3) | **0.009** |  |  |
|  | Placebo | 1.22 (0.78, 1.91) |  |  |  |  |
| 7S,14S-diHDPA | 300 mg | 0.84 (0.67, 1.07) | -8.8 (-34.8, 27.6) | 0.59 | - | - |
|  | 100 mg | 1.33 (1.05, 1.69) | 44.0 (2.9, 101.5) | 0.034 |  |  |
|  | Placebo | 0.93 (0.73, 1.18) |  |  |  |  |
| RvE1 | 300 mg | 0.79 (0.45, 1.38) | -24.6 (-66.3, 68.9) | 0.49 | - | - |
|  | 100 mg | 0.90 (0.51, 1.59) | -13.3 (-61.3, 94.0) | 0.73 |  |  |
|  | Placebo | 1.04 (0.59, 1.85) |  |  |  |  |
| RvE2 | 300 mg | 0.93 (0.76, 1.13) | -1.0 (-25.7, 31.8) | 0.94 | - | - |
|  | 100 mg | 1.00 (0.82, 1.23) | 7.1 (-19.6, 42.6) | 0.64 |  |  |
|  | Placebo | 0.94 (0.76, 1.15) |  |  |  |  |
| RvE3 | 300 mg | 1.40 (0.58, 3.40) | 37.8 (-60.9, 385.8) | 0.61 | - | - |
|  | 100 mg | 1.95 (0.80, 4.72) | 91.3 (-45.7, 574.4) | 0.31 |  |  |
|  | Placebo | 1.02 (0.42, 2.50) |  |  |  |  |
| LXA_4_ | 300 mg | 1.41 (0.79, 2.54) | 92.0 (-16.6, 341.8) | 0.12 | - | - |
|  | 100 mg | 1.30 (0.73, 2.34) | 76.9 (-23.1, 307.1) | 0.18 |  |  |
|  | Placebo | 0.74 (0.41, 1.33) |  |  |  |  |
| LXB_4_ | 300 mg | 0.36 (0.16, 0.82) | -54.6 (-85.9, 46.6) | 0.18 | - | - |
|  | 100 mg | 0.96 (0.42, 2.19) | 21.2 (-62.5, 291.4) | 0.75 |  |  |
|  | Placebo | 0.79 (0.34, 1.83) |  |  |  |  |
| 5S,15S-diHETE | 300 mg | 0.71 (0.32, 1.57) | -27.1 (-76.5, 126.1) | 0.58 | - | - |
|  | 100 mg | 0.52 (0.23, 1.15) | -46.8 (-82.9, 64.9) | 0.27 |  |  |
|  | Placebo | 0.98 (0.44, 2.18) |  |  |  |  |
| 13,14-dehydro-15-oxo-LXA_4_ | 300 mg | 0.83 (0.44, 1.55) | -30.5 (-71.7, 70.5) | 0.42 | - | - |
|  | 100 mg | 1.38 (0.74, 2.60) | 16.1 (-52.7, 185.0) | 0.74 |  |  |
|  | Placebo | 1.19 (0.63, 2.26) |  |  |  |  |
| 15-oxo-LXA_4_ | 300 mg | 0.85 (0.50, 1.46) | -19.2 (-62.3, 73.0) | 0.58 | - | - |
|  | 100 mg | 0.60 (0.35, 1.02) | -43.7 (-73.7, 20.5) | 0.14 |  |  |
|  | Placebo | 1.06 (0.62, 1.82) |  |  |  |  |
| 15-epi-LXA_4_ | 300 mg | 0.70 (0.37, 1.32) | -41.4 (-76.1, 44.0) | 0.24 | - | - |
|  | 100 mg | 1.31 (0.69, 2.46) | 9.5 (-55.5, 169.0) | 0.84 |  |  |
|  | Placebo | 1.19 (0.63, 2.26) |  |  |  |  |
| 15-epi-LXB_4_ | 300 mg | 0.85 (0.41, 1.76) | -31.7 (-75.7, 91.9) | 0.47 | - | - |
|  | 100 mg | 0.79 (0.38, 1.64) | -36.6 (-77.4, 78.2) | 0.38 |  |  |
|  | Placebo | 1.25 (0.60, 2.61) |  |  |  |  |
| LTB_4_ | 300 mg | 0.53 (0.28, 1.01) | -71.2 (-88.5, -27.8) | **0.008** | -58.1 (-83.2, 4.2) | 0.061 |
|  | 100 mg | 1.26 (0.66, 2.40) | -31.2 (-72.5, 72.3) | 0.42 |  |  |
|  | Placebo | 1.83 (0.95, 3.52) |  |  |  |  |
| 5S,12S-diHETE | 300 mg | 0.56 (0.30, 1.06) | -50.8 (-80.2, 22.1) | 0.12 | - | - |
|  | 100 mg | 0.72 (0.38, 1.36) | -36.8 (-74.5, 56.9) | 0.32 |  |  |
|  | Placebo | 1.13 (0.59, 2.17) |  |  |  |  |
| 20-OH-LTB_4_ | 300 mg | 0.77 (0.48, 1.24) | 30.4 (-34.0, 157.7) | 0.44 | - | - |
|  | 100 mg | 1.04 (0.65, 1.68) | 77.2 (-10.3, 250.1) | 0.099 |  |  |
|  | Placebo | 0.59 (0.36, 0.95) |  |  |  |  |
| 20-COOH-LTB_4_ | 300 mg | 1.09 (0.76, 1.55) | 11.5 (-33.0, 85.3) | 0.67 | - | - |
|  | 100 mg | 0.71 (0.50, 1.02) | -26.9 (-56.0, 21.5) | 0.22 |  |  |
|  | Placebo | 0.98 (0.68, 1.40) |  |  |  |  |
| PGE_2_ | 300 mg | 0.29 (0.18, 0.47) | -85.5 (-92.6, -71.5) | **<0.001** | -14.2 (-56.2, 67.8) | 0.65 |
|  | 100 mg | 0.34 (0.21, 0.55) | -83.1 (-91.4, -66.8) | **<0.001** |  |  |
|  | Placebo | 2.02 (1.25, 3.27) |  |  |  |  |
| PGD_2_ | 300 mg | 0.77 (0.48, 1.24) | -58.7 (-78.9, -18.9) | 0.011 | 5.7 (-45.8, 106.4) | 0.87 |
|  | 100 mg | 0.73 (0.45, 1.17) | -60.9 (-80.1, -23.3) | **0.007** |  |  |
|  | Placebo | 1.86 (1.15, 3.01) |  |  |  |  |
| PGF_2a_ | 300 mg | 1.01 (0.59, 1.72) | -44.1 (-73.9, 19.6) | 0.13 | - | - |
|  | 100 mg | 0.68 (0.40, 1.16) | -62.3 (-82.4, -19.3) | 0.013 |  |  |
|  | Placebo | 1.80 (1.05, 3.10) |  |  |  |  |
| TxB_2_ | 300 mg | 0.10 (0.04, 0.23) | -94.6 (-98.3, -82.9) | **<0.001** | 145.6 (-22.4, 677.3) | 0.13 |
|  | 100 mg | 0.04 (0.02, 0.09) | -97.8 (-99.3, -93.0) | **<0.001** |  |  |
|  | Placebo | 1.87 (0.82, 4.28) |  |  |  |  |

**Supplementary Table 6 Legend:** The mean fold-change and mean fold-change percent from baseline is shown for the three study arms for each lipid mediator. Analysis of variance were conducted to test the effect of the 100 mg and 300 mg dose of aspirin as compared to the placebo.

**References**:

1. O'Brien MP, Hunt PW, Kitch DW, et al. A Randomized Placebo Controlled Trial of Aspirin Effects on Immune Activation in Chronically Human Immunodeficiency Virus-Infected Adults on Virologically Suppressive Antiretroviral Therapy. *Open Forum Infect Dis.* 2017;4(1):ofw278.

2. Kaslow RA, Ostrow DG, Detels R, Phair JP, Polk BF, Rinaldo CR, Jr. The Multicenter AIDS Cohort Study: rationale, organization, and selected characteristics of the participants. *American journal of epidemiology.* 1987;126(2):310-318.

3. Barkan SE, Melnick SL, Preston-Martin S, et al. The Women's Interagency HIV Study. WIHS Collaborative Study Group. *Epidemiology.* 1998;9(2):117-125.

4. D'Souza G, Bhondoekhan F, Benning L, et al. Characteristics of the MACS/WIHS Combined Cohort Study: Opportunities for Research on Aging With HIV in the Longest US Observational Study of HIV. *American journal of epidemiology.* 2021;190(8):1457-1475.

**Supplementary Figures Title and Legend**

**Supplementary Figure 1: SPM metabolome and mediator abundance bar plot**

Supplementary Figure 1A: SPM metabolome abundance bar plot

Supplementary Figure 1B: SPM lipid mediator abundance bar plot

Supplementary Figure 1 Legend: Abundance heat map for SPM metabolome (A, left panel) and lipid mediator (B, right panel) are shown as a stacked bar graphs. Metabolome and individual mediators were log_10_ transformation and standardization. Color coding shows HIV status as well as the metabolome/mediator group.

**Supplementary Figure 2**: **Metabolome correlation plot**

Supplementary Figure 2 Legend: Two latent variables were selected based on PLS-DA. For each of the two latent variables, Pearson correlation coefficients were calculated for each of the 12 metabolomes as well as the cohort variable. Metabolomes with VIP>1 and positively associated with PWH are plotted in red and those with VIP>1 and negatively associated with PWH are plotted in blue.

**Supplementary Figures 3-5**: **PCA plot of metabolites by CD4 count and ART status**

Supplementary Figure 3-5 Legend: Principal components analysis (PCA) plots based on the lipid mediators are shown for the first 4 principal components (PC1-4). Color coding is done by CD4 T-cell count (Suppl Fig 3, CD4<500 and CD4>500), by INSTI ART regimen (Suppl Fig 4, INSTI yes vs. no) and PI ART regiment (Suppl Fig 5, PI yes vs. no).

**Supplementary Figure 6**: **Lipid mediator correlation plot**

Supplementary Figure 3 Legend: Two latent variables were shown based on PLS-DA. For each of the two latent variables, Pearson correlation coefficients were calculated for each of the 50 lipid mediators as well as the cohort variable. Mediators with VIP>1 and positively associated with PWH are plotted in red and those with VIP>1 and negatively associated with PWH are plotted in blue.
